# The SlyD metallochaperone targets iron-sulfur biogenesis pathways and the TCA cycle

**DOI:** 10.1128/mbio.00967-23

**Published:** 2023-08-16

**Authors:** Milica Denic, Evelyne Turlin, Deborah B. Zamble, Jean-Michel Betton, Daniel Vinella, Hilde De Reuse

**Affiliations:** 1 Unité Pathogenèse de Helicobacter, Département de Microbiologie, UMR CNRS 6047, Institut Pasteur, Université Paris Cité, Paris, France; 2 Université Paris Diderot, Sorbonne Paris Cité, Cellule Pasteur, Paris, France; 3 Department of Chemistry, University of Toronto, Toronto, Ontario, Canada; 4 Department of Biochemistry, University of Toronto, Toronto, Ontario, Canada; 5 Unité Adaptation au stress et Métabolisme chez les entérobactéries, Département de Microbiologie, UMR CNRS 6047, Institut Pasteur, Université Paris Cité, Paris, France; Rutgers The State University of New Jersey, Piscataway, New Jersey, USA

**Keywords:** protein chaperone, PPIase, *Helicobacter pylori*, Fe-S clusters, TCA cycle, antibiotic susceptibility, ROS susceptibility, metallochaperone, *Escherichia coli*

## Abstract

Control of protein folding is a conserved function in all domains of life. The ubiquitous peptidyl-prolyl *cis-trans* isomerases (PPIases) catalyze the *cis*/*trans*-isomerization of peptidyl-prolyl bonds, often a rate-limiting step for protein folding. PPIases are involved in central processes in eucaryotes but their functions in bacteria are poorly understood. We applied a large-scale two-hybrid screen to identify novel targets of SlyD, a metallochaperone PPIase, from the model organism *Escherichia coli*, and the important gastric pathogen *Helicobacter pylori*. SlyD from both organisms interacts with metal-containing proteins including TCA cycle enzymes and with enzymes driving the biogenesis of iron-sulfur (Fe-S) clusters. In *H. pylori*, a ∆*slyD* mutant contains strongly diminished amounts of MisSU, its sole Fe-S biogenesis proteins as well as diminished activity of the Fe-S aconitase. *E. coli* and *H. pylori ∆slyD* mutants are less susceptible to ampicillin and gentamicin antibiotics and under microaerobic conditions to the ROS-producing molecule hypochlorite. In addition, the intracellular ATP content and proton motive force are reduced in the *H. pylori* ∆*slyD* mutant, and its gentamicin and hypochlorite susceptibility phenotypes are rescued upon restoration of MisSU expression. Measurement of malate and fumarate in the *H. pylori ∆slyD* mutant suggests that the two enzymes before and after fumarase in the TCA cycle are affected. We conclude that SlyD regulates the activity of TCA cycle enzymes by controlling the efficiency of their Fe-S cluster formation. This is the first report of the role of a bacterial PPIase in central metabolism and susceptibility to stress and antibiotics.

**IMPORTANCE:**

Correct folding of proteins represents a crucial step for their functions. Among the chaperones that control protein folding, the ubiquitous PPIases catalyze the *cis*/*trans*-isomerization of peptidyl-prolyl bonds. Only few protein targets of PPIases have been reported in bacteria. To fill this knowledge gap, we performed a large-scale two-hybrid screen to search for targets of the *Escherichia coli* and *Helicobacter pylori* SlyD PPIase-metallochaperone. SlyD from both organisms interacts with enzymes (i) containing metal cofactors, (ii) from the central metabolism tricarboxylic acid (TCA) cycle, and (iii) involved in the formation of the essential and ancestral Fe-S cluster cofactor. *E. coli* and *H. pylori ∆slyD* mutants present similar phenotypes of diminished susceptibility to antibiotics and to oxidative stress. In *H. pylori*, measurements of the intracellular ATP content, proton motive force, and activity of TCA cycle proteins suggest that SlyD regulates TCA cycle enzymes by controlling the formation of their indispensable Fe-S clusters.

## INTRODUCTION

Protein folding is a controlled process in all cells. Among the conserved enzymes controlling this essential step, the ubiquitous peptidyl-prolyl *cis-trans* isomerase (PPIase) activity occupies a unique position. Indeed, these enzymes specifically catalyze the *cis*/*trans*-isomerization of peptidyl-prolyl (XAA-Pro) bonds in polypeptide chains, a reaction that occurs spontaneously in the cells but is often a rate-limiting step for protein folding. PPIases are required for the folding of some newly synthesized proteins and regulate the stability, localization, and activity of mature proteins (1). While their role in the eucaryotic immune response, neuronal differentiation, cell cycle control, and viral response is widely documented (2), the function of PPIases in bacteria and archaea is still poorly defined.

The SlyD PPIase is ubiquitous in bacteria (3). SlyD is a member of the FK506-binding protein (FKBP) subfamily characterized by the insertion of a chaperone domain into the FKBP region, which enables it to also function as an efficient molecular chaperone (4). A unique feature of SlyD proteins is a C-terminal extension varying from 2 to 50 residues in length that is rich in histidine and cysteine residues and binds divalent cations such as Ni^2+^, Zn^2+^, and Co^2+^. In *Escherichia coli*, the extension contains 13 histidine and six cysteine residues and was shown to bind up to seven nickel ions per SlyD molecule (5). Notably, nickel binding to the C-terminal region has been shown to downregulate the *in vitro* enzymatic activity of the *E. coli* SlyD PPIase (6).

The SlyD protein was originally discovered in *E. coli* as a host factor required for the function of the *φ*X174 bacteriophage E-protein (7). Since its discovery, finding *in vivo* SlyD targets and phenotypes associated with SlyD inactivation has proven to be difficult. This is probably because SlyD is usually not essential for the activity of its targets. Accordingly, the *E. coli ∆slyD* mutant has no growth defect. Importantly, most of the SlyD functions identified so far are associated with metal-related functions (8). In *E. coli*, the SlyD protein is required for nickel insertion and maturation of [NiFe]-hydrogenase, a process that depends on the SlyD C-terminal nickel-binding region and on the interaction between the SlyD chaperone domain and the hydrogenase accessory factor HypB (9). In addition, *E. coli* SlyD was recently found to participate in an YdiV-dependent regulation of the Fur regulator (10).

We have recently discovered that the SlyD protein of the major pathogen *Helicobacter pylori* is essential for colonization of a mouse model and is involved in the control of the uptake of nickel, an essential virulence determinant (11). *H. pylori* is a Gram-negative bacterium, discovered only 40 years ago, that belongs to the *Campylobacterota* phylum (formerly *Epsilonproteobacteria*) (12). *H. pylori* colonizes the stomach of about half of the human population worldwide. Upon infection, *H. pylori* establishes persistent gastric colonization leading to the development of gastritis and, in about 10% of the infected individuals, peptic ulcer disease (13, 14). In 1–3% of the infected people, decades of infection result in the development of gastric cancer, a very aggressive adenocarcinoma, causing up to 800,000 deaths worldwide every year. *H. pylori* is till now the sole bacterium recognized by IARC as a class I carcinogen, that is, causing cancer in humans (15).

Efficient and controlled nickel uptake, trafficking, and homeostasis is crucial for *H. pylori* virulence since the survival of this organism in the acidic stomach relies on two nickel-containing enzymes, urease and [NiFe]-hydrogenase. These two enzymes are essential for gastric colonization and important for the virulence of the bacterium (16, 17). Urease catalyzes the hydrolysis of urea to ammonium, which serves as a buffer that allows *H. pylori* to resist the acidity of the stomach (18). The [NiFe]-hydrogenase is also essential for colonization by allowing the bacterium to utilize molecular hydrogen as an energy substrate (19). *H. pylori* possesses two sole nickel uptake systems, the NixA permease (20) and the essential NiuBDE ABC transporter, which we previously characterized (21). We established that the SlyD chaperone function is required for full activity of NiuD, the nickel permease of the Niu ABC system in *H. pylori* cells, probably by direct physical interaction (11). The *H. pylori* SlyD protein comprises a C-terminal metal-binding domain that contains five histidine and five cysteine residues (11). Purified *H. pylori* SlyD protein binds several divalent cations including nickel (3, 22), and its *in vitro* PPIase enzymatic activity is also repressed by nickel (11).

Early interatomic studies suggested that *H. pylori* SlyD is part of a complex comprising the UreA urease subunit and the hydrogenase maturation accessory protein HypB (22, 23). It was reported later that *H. pylori* SlyD binds, through its chaperone domain, to HypB and to the TAT signal peptide of the periplasmic HydA [NiFe]-hydrogenase subunit (3, 6). However, in our studies, we found that neither hydrogenase nor urease activity is affected in the *H. pylori* ∆*slyD* mutant and that a *∆slyD* mutant has no growth defect (11). Thus, although a pleiotropic function of SlyD in both *E. coli* and *H. pylori* is anticipated, very few was known on their targets and even less was reported on the phenotypes of *slyD* mutants. To fill this knowledge gap, we applied a large-scale two-hybrid screening strategy to identify new targets of the SlyD protein, both in *E. coli* and in *H. pylori*. In order to attribute novel functions to the SlyD protein in both organisms, we performed a phenotypic analysis of *E. coli* and *H. pylori* mutant deficient in the *slyD* protein and its newly identified targets.

We discovered that, in both bacteria, SlyD protein is interacting with iron-sulfur (Fe-S) biogenesis proteins and interfering with central carbon metabolism. Phenotypic analysis revealed a major role of SlyD in the response to antibiotics and oxidative stress of *E. coli* and *H. pylori*. While long overlooked, it is now accepted that, under certain circumstances, the bacterial metabolic state influences bacterial susceptibility to stress and particularly to antibiotics (24). Several physiological factors affecting the metabolic state are proposed to impact antibiotic resistance or persistence but only few effectors have been identified. Among these factors are toxin-antitoxin systems, (p)ppGpp or the SOS system, this latter being absent from *H. pylori*. Our present study identified, for the first time, a bacterial PPIase as a novel factor controlling central metabolic functions and susceptibility to stress and antibiotics.

## RESULTS

### Large-scale screen of *E. coli* and *H. pylori* SlyD interactors

To search for interactors of SlyD from *E. coli* and *H. pylori*, we performed a large-scale screen using bacterial two hybrid (BACTH) experiment (25). *Ec-*SlyD and *Hp-*SlyD were fused at their C- and N-terminus to the T25 adenylate cyclase fragment into pKT25 and pNKT25 vectors, respectively, and transformed in the *E. coli* strain DHM1. These strains were then electro-transformed by an *E. coli* or an *H. pylori* whole genomic library randomly cloned into vector pUT18C expressing the T18 adenylate cyclase fragment. On plates containing X-gal, the blue colonies attesting to the reconstitution of the adenylate cyclase activity were selected and the inserts of pUT18C were sequenced. For the screen with *Ec*SlyD, only five positive clones with inserts in frame with the T18 encoding sequence were obtained (Table S1). For the *Hp*SlyD, among the 22 clones that we sequenced, only nine contained an open reading frame in frame with the T18 gene (Table S2).

### SlyD interacts with proteins with metal co-factors in both *E. coli* and *H. pylori*

**Fig 1:**
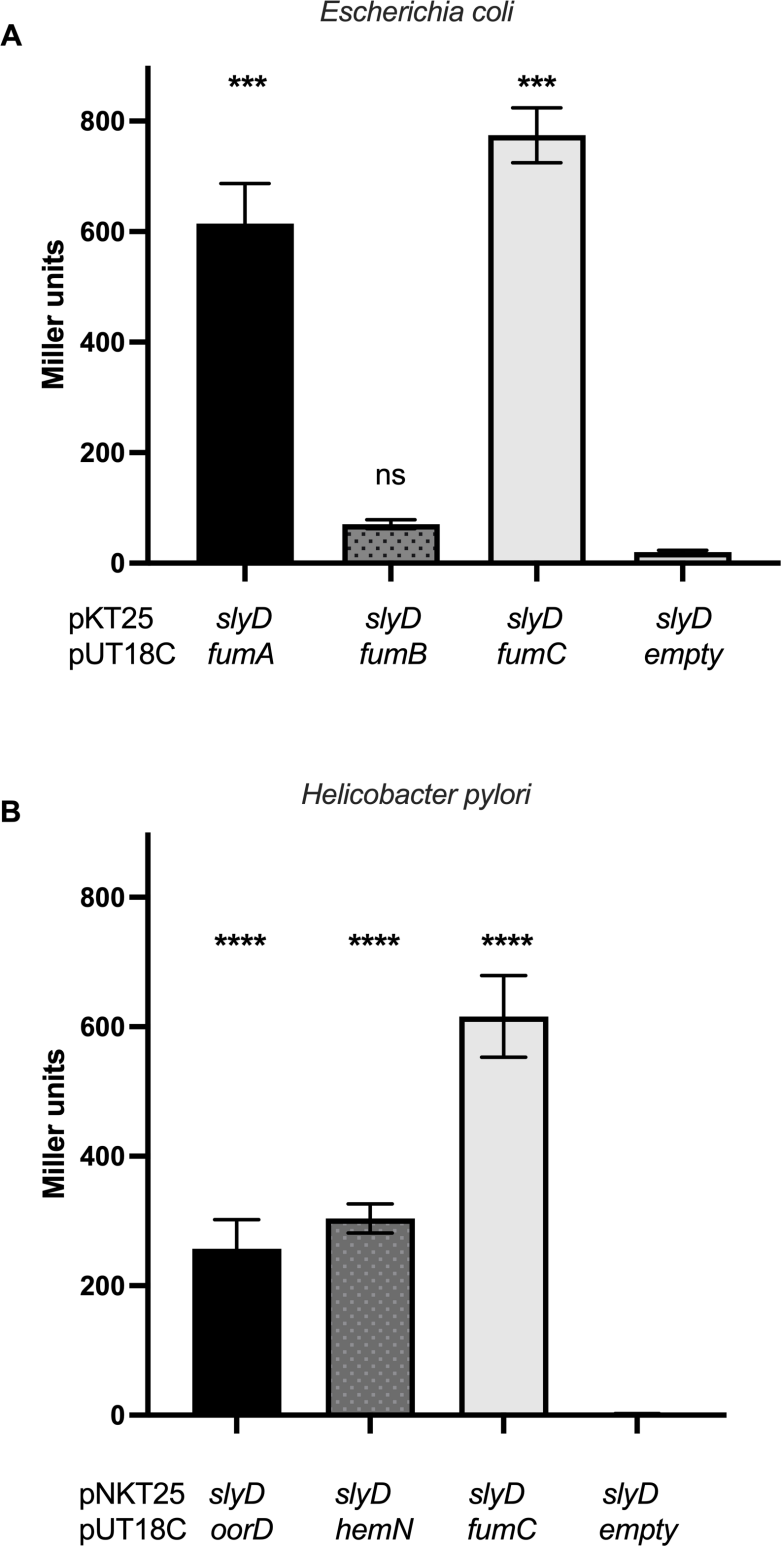
BACTH pairwise tests of interactions of the *Escherichia coli* and *Helicobacter pylori* SlyD proteins. (A) β-Galactosidase activities of pairwise combinations of the *E. coli* SlyD protein with FumA, FumB, and FumC proteins. (B) β-Galactosidase activities of pairwise combinations of the *H. pylori* SlyD protein with OorD, HemN, and FumC proteins. β-Galactosidase activities are expressed in Miller units (29). Each measurement corresponds to at least three independent experiments and at least five replicates. Error bars represent the standard deviation. *** and **** correspond to the *P* values ≤0.001 and ≤0.0001, in comparison with the *slyD*-empty control activity, respectively. ns corresponds to nonsignificant (*P* > 0.05).

Among the hits of our SlyD interactors screen, fumarase was detected in both *E. coli* and *H. pylori*. For the *H. pylori* hits, six out of the nine interactors had a metallic cofactor (Zn, Fe, Mg, Fe-S clusters) (Table S2). In order to individually validate and extend the results of the screens, we first tested the interactions of *Ec*SlyD and *Hp*SlyD with fumarases (Fig. 1). Fumarases (or fumarate hydratases) catalyze the reversible hydration of fumarate to malate and take part in the tricarboxylic acid (TCA) cycle. *E. coli* possesses three fumarases: FumA, a class I fumarate hydratase that relies on a Fe_4_S_4_ cluster for its activity and functions under aerobic conditions. FumB also contains a Fe_4_S_4_ cluster and is mainly expressed during anaerobic growth (26, 27). FumC is a class II fumarate hydratase, which is iron-independent and functions as a backup under iron limitation and oxidative stress conditions. *H. pylori* possesses a sole FumC fumarase (28). Using BACTH, we measured robust interactions of the *Ec*SlyD with *Ec*FumA and with *Ec*FumC while no interaction was found with FumB (Fig. 1A). The interaction of *Hp*SlyD with *Hp*FumC and with two Fe_4_S_4_ cluster proteins, OorD and HemN, was also validated (Fig. 1B). OorD is the delta subunit of the 2-oxoglutarate ferrodoxin oxidoreductase. This enzyme contains a Fe_4_S_4_ cluster and is also part of the TCA cycle. HemN also containing an Fe_4_S_4_ cluster is an oxygen-independent coproporphyrinogen III oxidase involved in the biosynthesis of heme, an iron-containing cofactor of enzymes involved in energy metabolism, detoxification, and sensing of environmental cues. These results point to an interaction between the SlyD protein and proteins containing metal co-factors, in particular Fe-S clusters, both in *E. coli* and in *H. pylori*.

### SlyD interacts with proteins of the Fe-S biogenesis pathways in *E. coli* and *H. pylori*

Using tandem affinity purification (TAP) in *H. pylori*, we previously defined the interactome of the hydrogenase accessory protein HypB and found that it includes both the SlyD protein and the two sole Fe-S biogenesis proteins of this bacterium (23). Fe-S clusters are made and delivered to apo-proteins by dedicated machineries of different types but working on the same principle: a cysteine desulfurase produces sulfur from L-cysteine, iron and sulfur meet and form a cluster on scaffold proteins, then carrier proteins deliver the cluster to terminal apo-targets (30). By analogy with the Fe-S machinery of nitrogenase-containing bacteria, the two sole *H. pylori* proteins were originally designated NifU-NifS, despite the absence of nitrogenase in this organism (31). This inconsistency has recently been solved by a large context and phylogeny analysis revealing that the two Fe-S machinery proteins of *H. pylori* belong to a specific class of “minimal Fe-S biogenesis systems,” accordingly renamed MisU and MisS for a cysteine desulfurase and a scaffold protein, respectively (32, 33). In contrast, *E. coli* possesses two dedicated machineries, ISC and SUF, which synthesize and deliver Fe-S clusters to their cognate apo-proteins (30).

**Fig 2:**
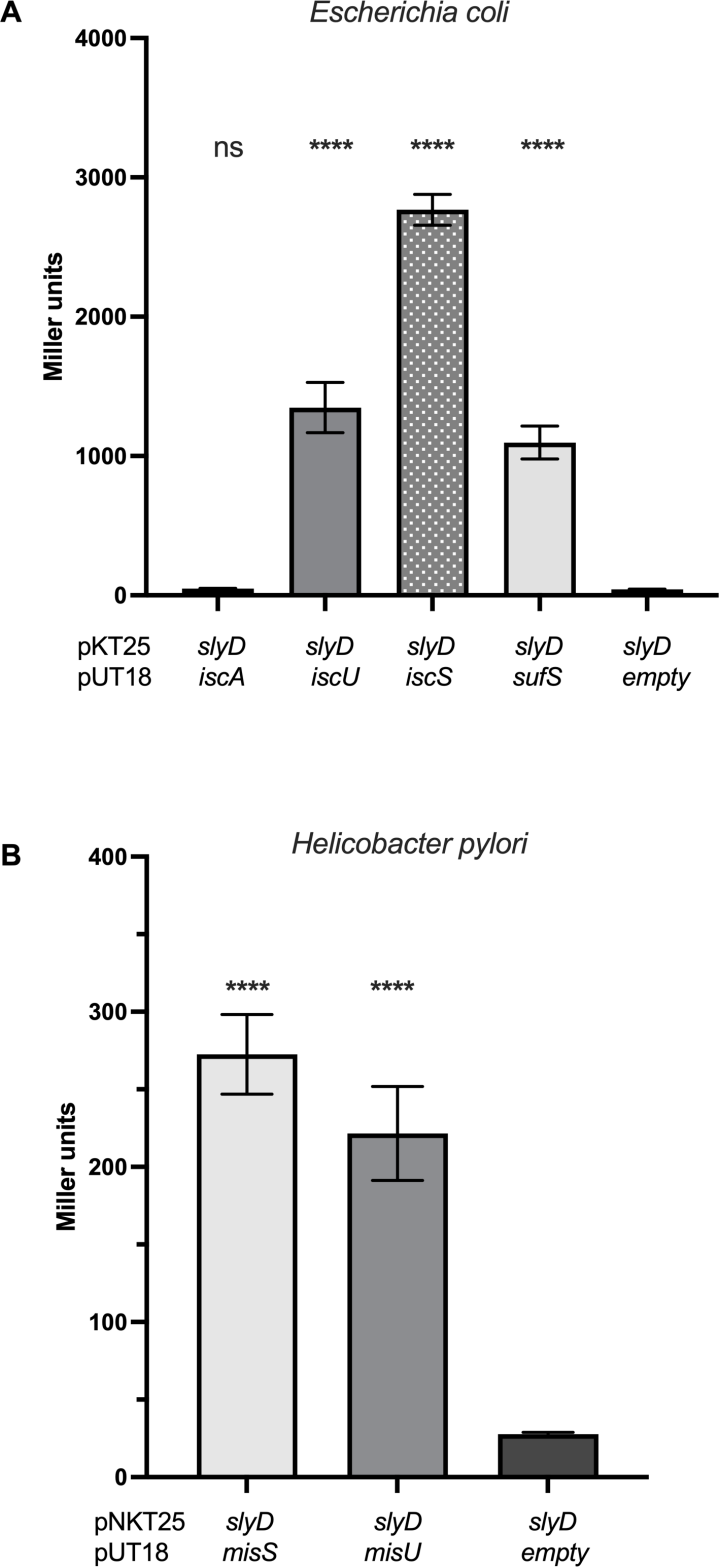
The SlyD proteins of *Escherichia coli* and *Helicobacter pylori* interact with major proteins of the iron-sulfur cluster biogenesis machineries. (A) β-Galactosidase activities of pairwise combinations of the *E. coli* SlyD protein with IscA, IscU, IscS, and SufS proteins, tested by BACTH. (B) β-Galactosidase activities of pairwise combinations of the *H. pylori* SlyD protein with MisS and MisU proteins, tested by BACTH. β-Galactosidase activities are expressed in Miller units (29). Each measurement corresponds to at least three independent experiments and at least five replicates. Error bars represent the standard deviation. *** and **** correspond to *P* values ≤0.001 and ≤0.0001, in comparison with the *slyD*-empty control activity, respectively. ns corresponds to nonsignificant (*P* > 0.05).

Thus, our TAP data together with the present BACTH results prompted us to systematically test the interactions of SlyD with the proteins involved in Fe-S biogenesis in both *E. coli* and *H. pylori* (Fig. 2). Most interestingly, we found that *Ec*SlyD interacts with core components of both the ISC and SUF machineries, namely with *Ec*IscU, *Ec*IscS, and *Ec*SufS, while *Ec*SlyD presented an interaction with neither *Ec*IscA nor *Ec*SufA, B, or C.

For *H. pylori*, our BACTH experiments demonstrated the interaction of *Hp*SlyD with both MisU and MisS proteins. These data indicate that SlyD interacts with Fe-S cluster biogenesis proteins in both *E. coli* and *H. pylori*.

### Does SlyD impact Fe-S synthesis machinery or Fe-S proteins?

To examine whether SlyD could play a role in the biogenesis of Fe-S clusters, three strategies were applied. First, in *E. coli*, we analyzed the maturation of two regulatory proteins, whose activities depend on the insertion of Fe-S centers, IscR (Fe_2_S_2_) and NsrR (Fe_4_S_4_). To this end, we measured the activity of target promoters of these regulators using the reporter fusions P*iscR-lacZ* and P*hmpA-lacZ*, as previously published (34) (Table S3). Transcription of the P*iscR-lacZ* and P*hmpA-lacZ* fusions is repressed by the holo-forms of IscR-Fe_2_S_2_ and NsrR-Fe_4_S_4_, respectively. IscR is maturated by the ISC machinery only, whereas NsrR can be maturated by ISC and SUF. No change in the expression of the *lacZ* fusions was measured in the *E. coli* ∆*slyD*-∆*iscAU* and ∆*slyD*-∆*suf* mutants as compared to the wild-type (WT) strain indicating that the activity of SlyD is not required for full Fe-S maturation of IscR and NsrR (data not shown).

**Fig 3:**
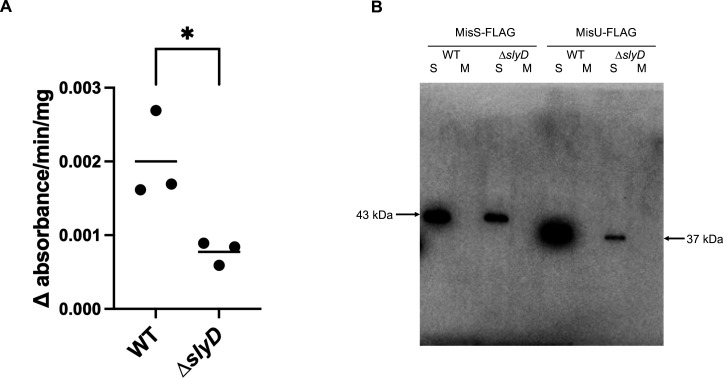
Test of the influence of SlyD on the Fe-S cluster biogenesis machinery in *H. pylori*. (A) Assay of the activity of the Fe-S cluster containing aconitase enzyme of *H. pylori* wild type and ∆*slyD* mutant. The aconitase activity is expressed as the ∆ of absorbance at 240 nm/min/mg of protein. The results of three independent assays are presented by dots. The mean is indicated by a bar. * corresponds to a *P*value of ≤0.05 with the Wilcoxon test. (B) Western blot of MisS-FLAG and MisU-FLAG proteins, the sole two Fe-S cluster biogenesis proteins of *H. pylori* in a wild-type strain and the *∆slyD* mutant, soluble (**S**) and membrane (**M**) extracts are shown. Anti-FLAG antibody was used. Calibration of the protein loading of each lane was performed with the stain-free image of the gel used for western blot (Fig. **S1**). This western has been reproduced three times with identical results for the ratio between wild-type strain and the *∆slyD* mutant, and with two independent *misS-flag* and *misU-flag* expressing strains.

The second strategy was to measure whether the activity of the Fe-S-containing enzyme aconitase relies on the SlyD protein. This was tested in *H. pylori*, our main organism of interest that, in contrast to *E. coli*, expresses a sole aconitase B. When compared to the WT strain, the aconitase activity of the *H. pylori ∆slyD* mutant presented a 2.6-fold reduction (Fig. 3A).

As a third approach, we analyzed the amounts of the MisS and MisU proteins by western blot using *H. pylori* WT and *∆slyD* strains, expressing MisS-FLAG and MisU-FLAG tagged proteins, from their native locus. Fractionation confirmed that these two proteins are soluble. Interestingly, we reproducibly observed, in the *∆slyD* context as compared to the WT strain, a 3-fold and 18-fold reduction in the amounts of MisS-FLAG and MisU-FLAG, respectively (Fig. 3B and Fig. S1A). Using RT-qPCR, we could exclude a role of SlyD in the control of the *misS* and *misU* genes transcription as their expressions were similar in the WT and *∆slyD* mutant (Fig. S1B). These data show that, in *H. pylori*, WT amounts of the two sole Fe-S biogenesis proteins rely on an active SlyD protein.

### Both *E. coli ∆slyD* and *H. pylori ∆slyD* mutants present reduced susceptibility to ampicillin and gentamicin

**Fig 4:**
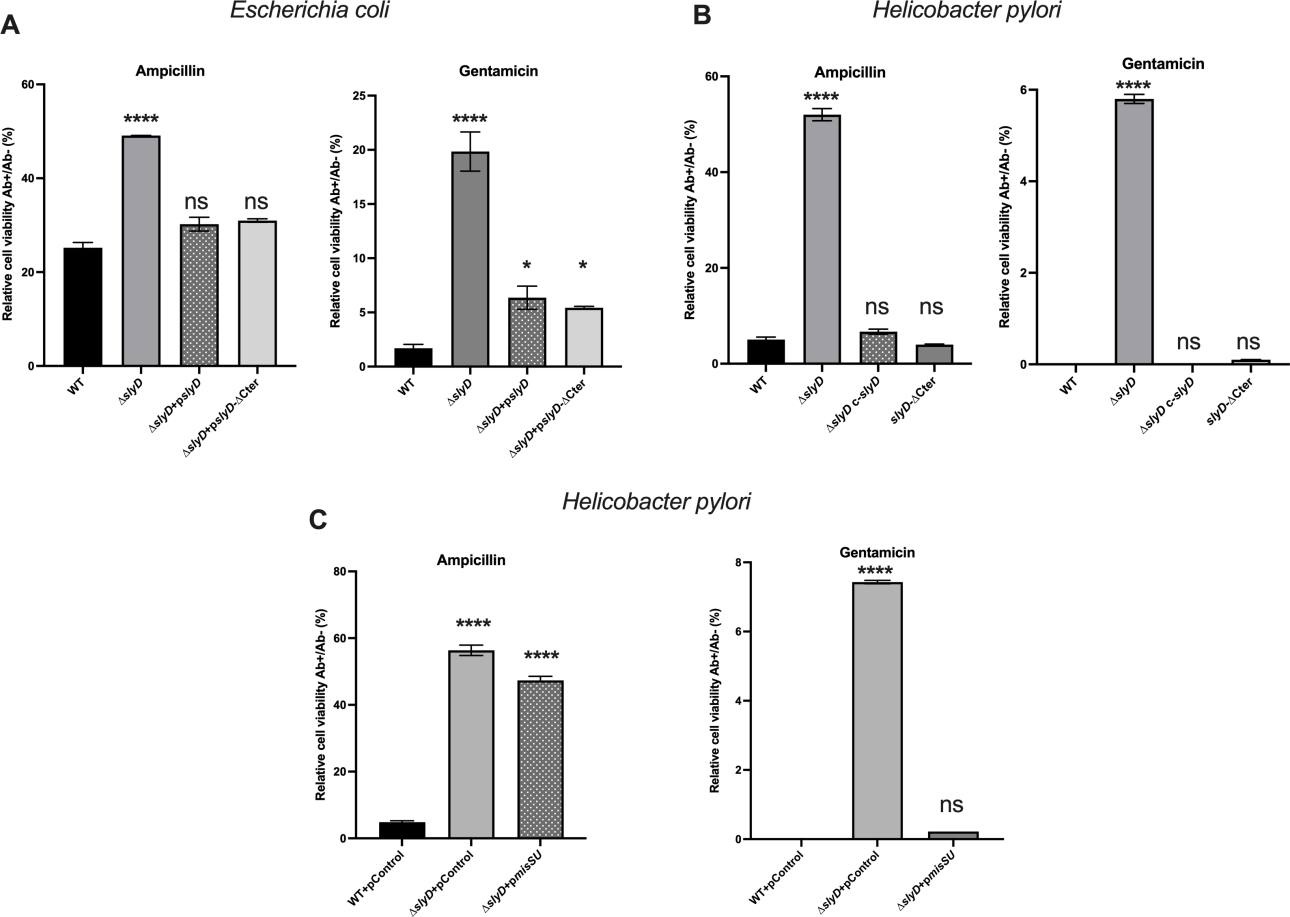
The *∆slyD Escherichia coli* mutant and the *∆slyD Helicobacter pylori* mutant present reduced susceptibility to ampicillin and gentamicin. (A) Susceptibility to ampicillin and gentamicin of *E. coli* wild type and mutants including a *∆slyD* mutant, a *∆slyD* mutant carrying a plasmid expressing wild-type SlyD (*pslyD*) or a *slyD-∆Cter* mutant. Experiments were performed in triplicate with ampicillin at 10 µg·mL^−1^ and gentamicin at 5 µg·mL^−1^. (B) Susceptibility to ampicillin and gentamicin of *H. pylori* wild-type strain, of a *∆slyD* mutant*,* of *∆slyD c-slyD*: a strain chromosomally complemented with a wild-type *slyD* gene and a *slyD*-∆C mutant. Experiments were performed in triplicate with ampicillin at 10 µg·mL^−1^ and gentamicin at 5 µg·mL^−1^. (C) Susceptibility to gentamicin of *H. pylori* wild-type strain and the *∆slyD* mutant with plasmids expressing *misSU* (p*misSU*) or a control plasmid (pcontrol). Experiments were performed in triplicate with gentamicin at 5 µg·mL^−1^. Relative cell viability corresponds to the percentage of colony-forming units (CFUs) with antibiotics versus without antibiotics. Error bars represent the standard deviation. The *P* values were calculated in comparison with the WT values, * corresponds to *P* ≤ 0.05, ** corresponds to *P* ≤ 0.01, *** corresponds to *P* ≤ 0.001, and **** corresponds to *P* ≤ 0.0001. ns corresponds to nonsignificant (*P* > 0.05).

Given the pleiotropic activity of SlyD and the established relation of Fe-S cluster enzymes and central metabolism with antibiotic resistance or tolerance (35), we decided to analyze this phenotype for the *∆slyD* mutants of *E. coli* and *H. pylori*. Cell viability was tested upon exposure to two antibiotics: gentamicin, an aminoglycoside that targets the ribosome, and ampicillin, a beta-lactam that inhibits the synthesis of peptidoglycan. Among the treatments used against *H. pylori* infections, aminoglycosides, including gentamicin, are not employed. On the contrary, the beta-lactam amoxicillin, but not ampicillin, is part of the classical treatment used against *H. pylori* in clinics. We found that the deletion of the *slyD* gene was associated with significantly decreased susceptibility to gentamicin and ampicillin, both in *E. coli* and *H. pylori*, in this latter organism it was particularly prominent (Fig. 4). This phenotype was not observed in the *slyD-∆C* mutant (deletion of the C-terminal region) indicating that it does not rely on the C-terminal domain. WT sensitivity to both antibiotics was recovered upon complementation, i.e., the re-introduction of WT *slyD* copies in *∆slyD* mutants of both organisms (Fig. 4). Thus, the SlyD protein is involved in susceptibility to two antibiotics in *E. coli* and *H. pylori*.

### Antibiotic susceptibility of mutants deleted in genes encoding SlyD interactors

**Fig 5:**
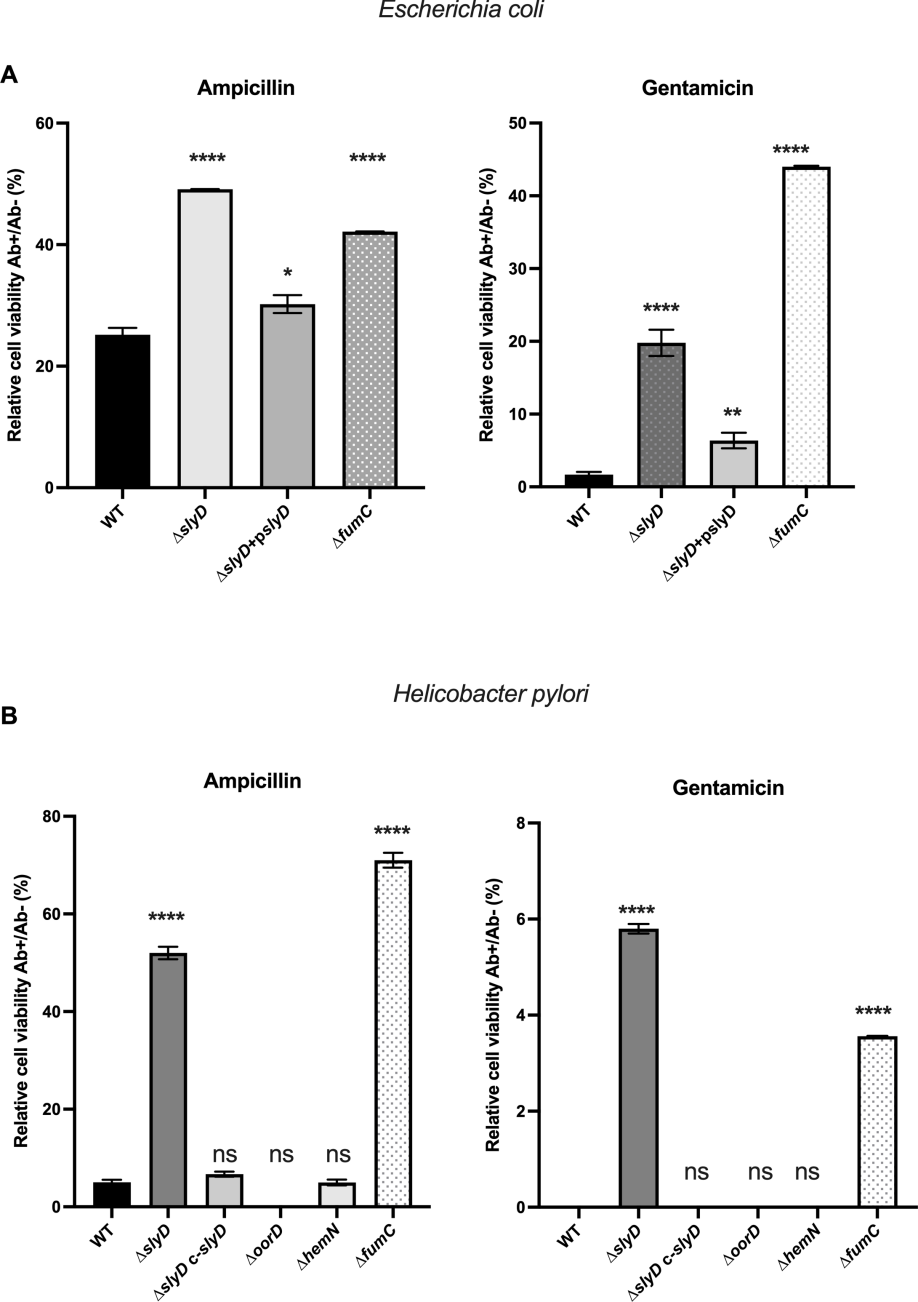
Susceptibility to ampicillin and gentamicin of mutants carrying deletions of genes encoding SlyD-interacting protein partners. (A) Susceptibility to ampicillin and gentamicin of *Escherichia coli* wild-type strain and the *∆fumC* mutant strains, *∆slyD* and ∆*slyD+pslyD* are shown for comparison. Experiments were performed in triplicate with ampicillin at 10 µg·mL^−1^ and gentamicin at 5 µg·mL^−1^. (B) Susceptibility to ampicillin and gentamicin of *H. pylori* wild type strain and the *∆fumC*, *∆oorD*, and *∆hemN* mutants, strains *∆slyD* and ∆*slyD c-slyD* are shown for comparison. Experiments were performed in triplicate with ampicillin at 10 µg·mL^−1^ and gentamicin at 5 µg·mL^−1^. Relative cell viability corresponds to the percentage of colony-forming units (CFUs) with antibiotics versus without antibiotics. Error bars represent the standard deviation. The *P* values were calculated in comparison with the WT values; * corresponds to *P* ≤ 0.05, ** corresponds to *P* ≤ 0.01, and **** corresponds to *P* ≤ 0.0001. ns corresponds to nonsignificant (*P* > 0.05).

We then asked whether the diminished antibiotic susceptibility of the *∆slyD* mutant could be mediated by one of its interactors. In *E. coli*, we observed that the *∆fumC* mutant was also less susceptible to the two antibiotics (Fig. 5A). In *H. pylori*, mutants carrying a total deletion of the *oorD*, *hemN* or *fumC* genes were easily obtained and did not present any significant growth defect indicating that these genes are not essential under normal conditions. As shown in Fig. 5B, only the *H. pylori ∆fumC* mutant presented a decreased sensitivity to the two tested antibiotics comparable to that of the *∆slyD* mutant. Thus, in both *E. coli* and *H. pylori,* deletion of a TCA cycle gene *fumC*, phenocopies the reduced susceptibility of the *∆slyD* mutant to antibiotics. The *misS* and *misU* genes being essential in *H. pylori*, their importance in this phenotype could not be evaluated. Given that the amounts of MisS and MisU proteins are reduced in the *H. pylori ∆slyD* mutant, we tested whether overexpression of these proteins from a plasmid could restore the antibiotic susceptibility. Indeed, overexpression of MisS-U in an *H. pylori ∆slyD* mutant rescues the susceptibility to gentamicin while it had no effect on ampicillin resistance. This suggests that, in *H. pylori*, the *∆slyD* gentamicin resistance phenotype is associated to reduced amounts of the sole Fe-S biogenesis proteins (Fig. 4C).

### SlyD participates in the *E. coli* and *H. pylori* response to oxidative stress

Several studies report a link between the bacterial response to oxidative stress and antibiotic susceptibility, although this was never analyzed in *H. pylori*. Thus, we examined how exposure to oxidative stress impacted the survival of *E. coli* and *H. pylori* ∆*slyD* mutants. Chronic infection by *H. pylori* induces a strong inflammatory response associated with neutrophil infiltration and the production of reactive oxygen species (ROS). In neutrophiles, hydrogen peroxide and superoxide are funneled into the production of hypochlorite (HOCl) as the major product, a molecule that also acts as a chemoattractant to *H. pylori* (36). Because the response of *H. pylori* to hypochlorite has been previously characterized and shown to be robust (36), this oxidative stress was tested for both organisms.

**Fig 6:**
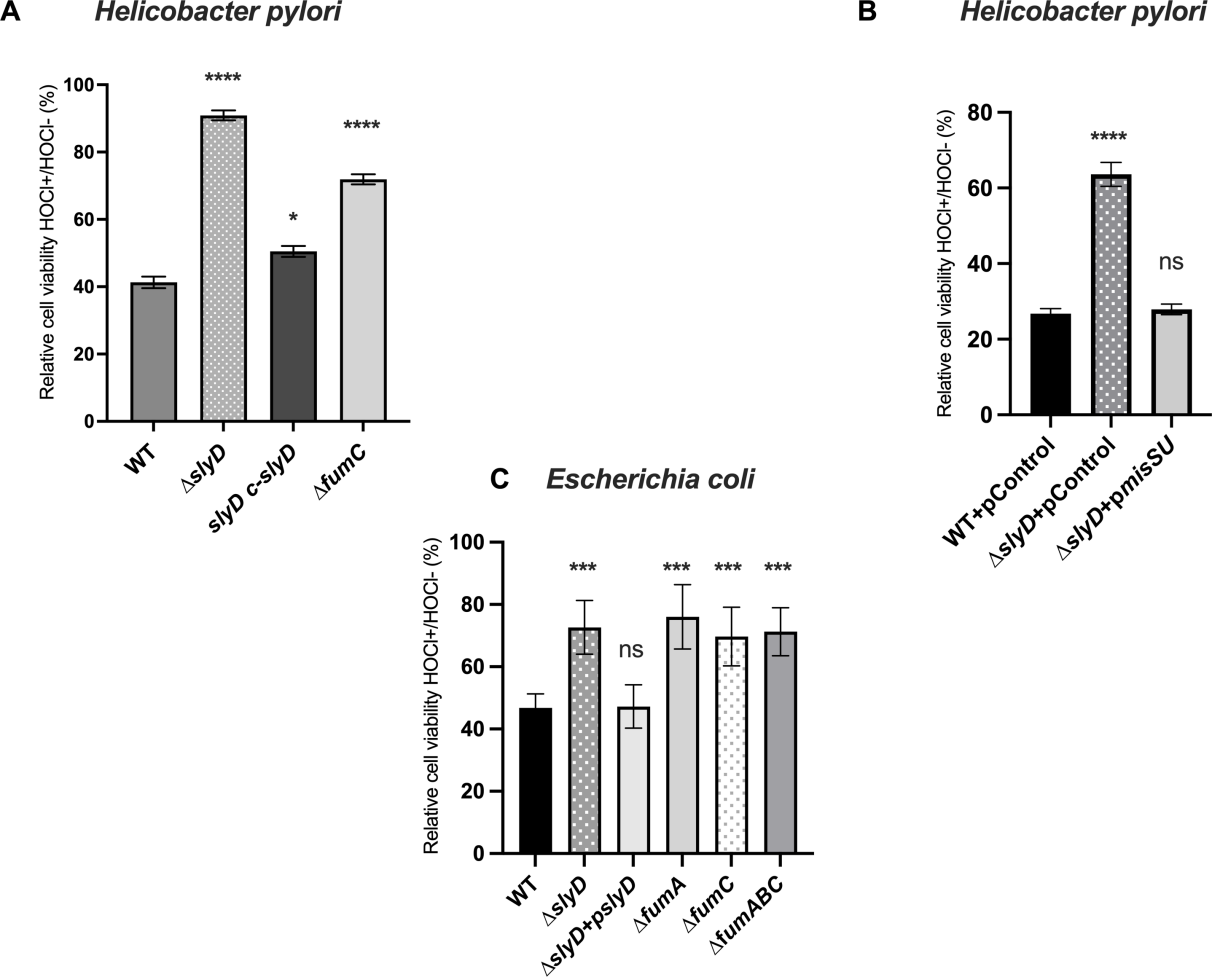
Susceptibility to oxidative stress of wild-type *E. coli* and *H. pylori* strains and of mutants. (A) Susceptibility to 1 mM hypochlorite (HOCl) of *H. pylori* under microaerobic conditions. The tested strains were wild type, *∆slyD*, ∆*slyD c-slyD,* and *∆fumC*. Experiments were performed in triplicate. (B) Susceptibility to hypochlorite of *H. pylori* wild-type strain and the *∆slyD* mutant with plasmids expressing *misSU* (p*misSU*) or a control plasmid (pControl). Experiments were performed in triplicate. (C) Susceptibility to 1.5 µM hypochlorite (HOCl) of *E. coli* under microaerobic conditions. The tested strains were wild type, *∆slyD*, ∆*slyD+pslyD*, ∆*fumA*, *∆fumC,* and ∆*fumABC*. Experiments were performed in triplicate. Relative cell viability corresponds to the percentage of colony-forming units (CFUs) with HOCl versus without addition. Error bars represent the standard deviation. The *P* values were calculated in comparison with the WT values; * corresponds to *P* ≤ 0.05, *** corresponds to *P* ≤ 0.001, and **** corresponds to *P* ≤ 0.0001. ns corresponds to nonsignificant (*P* > 0.05).

As *H. pylori* is a microaerophilic organism, the whole experiment was standardized under microaerobic conditions. To our surprise, we found that by measuring cell viability the *H. pylori ∆slyD* mutant is significantly more resistant to HOCl than the parental strain and the WT phenotype was restored in the *slyD* complemented strain (Fig. 6A). Moreover, WT sensitivity to hypochlorite was restored in the *∆slyD* mutant upon overexpression of the MisS-U proteins from a plasmid (Fig. 6B). In *E. coli*, a facultative anaerobe, the response to HOCl was tested during growth under different atmospheres. Only under microaerobic conditions, we found that the *E. coli ∆slyD* is also more resistant to the hypochlorite stress (Fig. 6C). Given the data of antibiotic resistance shown earlier, we also investigated the role of fumarase in the response of both organisms to HOCl. We found that both *E. coli* and *H. pylori ∆fumC* mutants as well as the *E. coli ∆fumA* mutant were more resistant to hypochlorite under microaerobic conditions.

### Measurement of the impact of the *slyD* and *fumC* mutations on the proton motive force and ATP concentration in *H. pylori*

**Fig 7:**
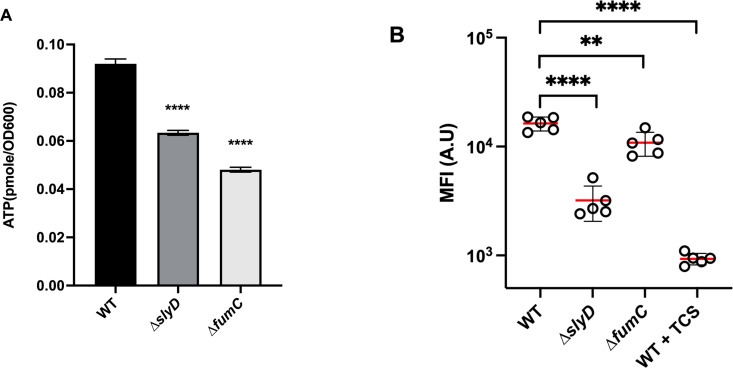
Measurement of intracellular ATP content and of the proton motive force in *H. pylori* wild-type strain and mutants. (A) Intracellular ATP content of *H. pylori* WT, *∆slyD,* and *∆fumC* cells was determined using a luciferase-based assay (BacTiter-Glo, Promega). Results from three independent experiments performed in triplicates are shown. Error bars represent the standard deviation. The *P*values were calculated in comparison with the WT values; ****, *P* ≤ 0.0001. (B) Proton motive force (pmf) measurements of *H. pylori* WT, *∆slyD,* and *∆fumC* cells. MitoTracker Red CMXRos, a membrane potential-reactive dye was used to analyze the samples of live *H. pylori* bacteria. Samples were stained with MitoTracker Red CMXRos and the PMF was measured by flow cytometry as the mean fluorescence intensity (MFI) in A.U. (arbitrary units). The cell population distribution histograms are presented in Fig. S2, for each condition, 50,000 cells were analyzed. As a control, the wild-type cells were treated with 500 µM TCS (3,3',4',5-tetrachlorosalicylanilide), a protonophore active on *H. pylori*, resulting in a massive loss of membrane potential. The experiment was performed three times. Student’s *t* test was used to determine significant differences of the means of the data with the WT cells. Error bars represent the standard deviation, with ** corresponding to *P* < 0.01 and **** corresponding to *P* < 0.0001, indicating that the mean values are significantly different from the wild-type mean value.

The activities of TCA cycle enzymes including fumarases are required for ATP synthesis and maintenance of the proton motive force (pmf). In both *∆slyD* and *∆fumC H. pylori* mutants, ATP concentration and pmf were measured using protocols that we previously validated in this organism (37). TCS (3,3′,4′,5-tetrachlorosalicylanilide), a protonophore active in *H. pylori* was used as a negative control for the pmf measurements. The results showed that in both the *H. pylori ∆slyD* and the *∆fumC* mutants, the intracellular ATP concentration was significantly diminished (Fig. 7A). For the pmf measurement by flow cytometry, a highly significant reduction was observed in the *∆slyD* mutant as compared to the parental strain while the pmf of the ∆*fumC* mutant was only mildly but significantly diminished (Fig. 7B and Fig. S2).

These data indicate that in an *H. pylori* mutant deficient in SlyD, the TCA cycle is indeed affected leading to a reduced cellular energetic status.

### SlyD is impacting the TCA cycle in *H. pylori*

Our data so far point to a role of SlyD in Fe-S biogenesis pathways and in the TCA cycle. FumC does not contain an Fe-S cluster, but being the sole fumarase it occupies a central position in the *H. pylori* TCA cycle. We decided to measure, in *H. pylori* WT strain and mutants, the intracellular concentrations of the substrate and product of the fumarase enzyme, namely fumarate and malate. Coupled enzymatic tests were applied for both metabolites using cell extracts of the WT strain and the *∆slyD* and *∆fumC* mutants. We also tested the effect of the addition of malate and fumarate to the cultures.

**Fig 8:**
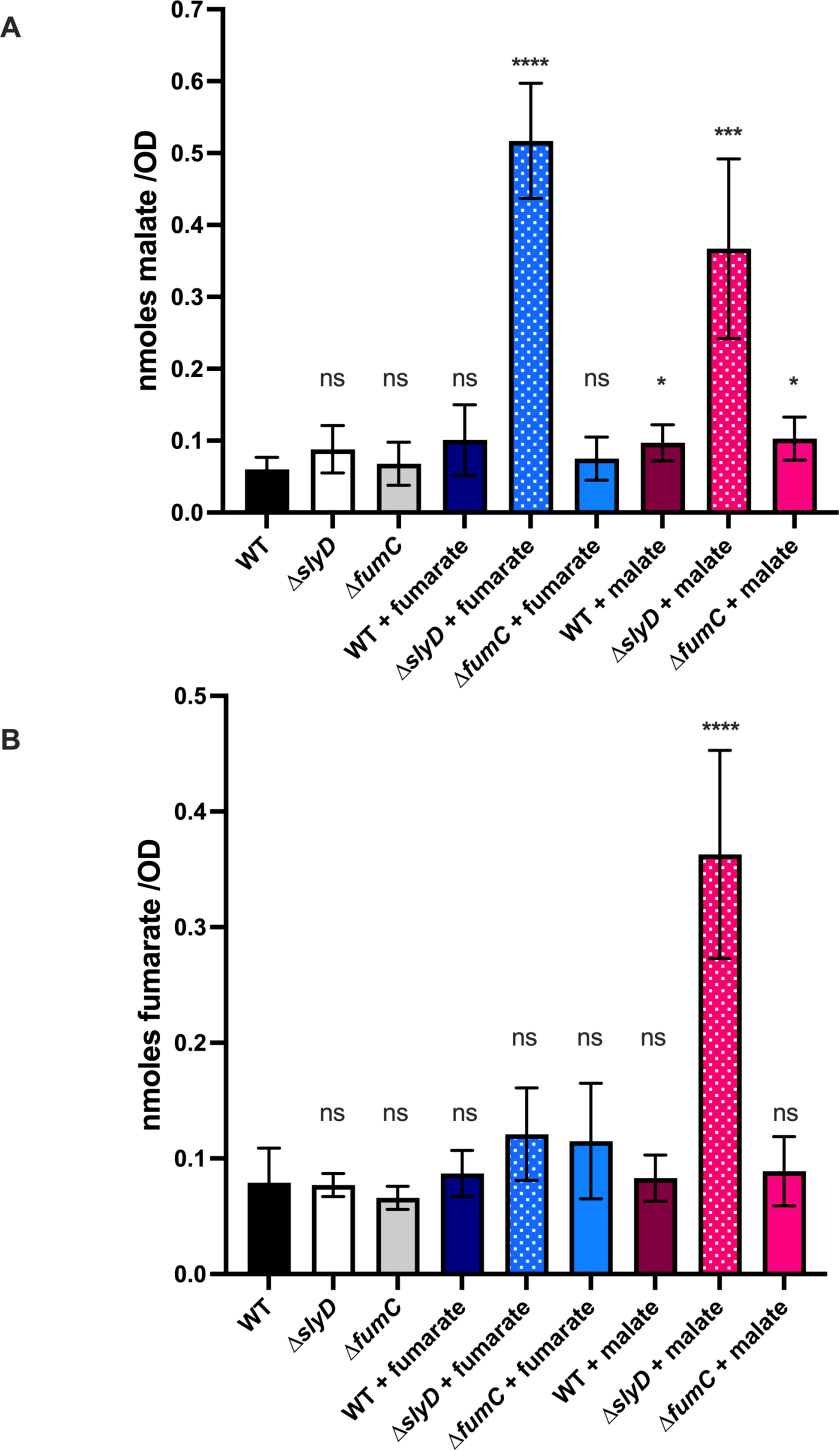
Measurement of intracellular concentrations of fumarate and malate in *H. pylori* wild-type strain and mutants. (A) Fumarate measurement of cells from *H. pylori* wild-type strain and *∆fumC*, *∆slyD* mutants grown with the addition of malate 5 mM of fumarate 25 mM or without any addition. (B) Malate measurement of cells from wild-type strain and *∆fumC*, *∆slyD* mutants grown with the addition of malate 5 mM of fumarate 25 mM or without any addition. Error bars represent the standard deviation. *, ***, and **** correspond to *P* values of ≤0.05, ≤0.001, and ≤0.0001, respectively, in comparison with the WT strain with any addition. ns corresponds to nonsignificant (*P* > 0.05). These experiments were performed three times in triplicate.

No major difference in the concentrations of both metabolites was observed for the *∆slyD* and *∆fumC* mutants grown under normal conditions (Fig. 8A and B). Upon the addition of nontoxic concentrations of malate (5 mM) and of fumarate (25 mM), striking differences in the metabolites’ concentrations were measured only in the *∆slyD* mutant. Indeed, in the presence of fumarate, the *∆slyD* mutant accumulates high concentrations of malate (8.5-fold that of the WT strain) (Fig. 8A and B). In the presence of malate, the *∆slyD* mutant accumulates both malate and fumarate, 6- and 4.7-fold more than that of the WT strain, respectively (Fig. 8A and B). We concluded that SlyD has a general impact on the TCA cycle, in particular on the enzymes “flanking” the FumC fumarase step, namely the malate:quinone oxidoreductase (MQO) and the fumarate reductase (FrdB). These two latter enzymes directly or indirectly depend on Fe-S clusters’ biogenesis; FrdB is an Fe_3_S_4_ enzyme and just like MQO relies, for its activity, on menaquinones, whose synthesis is Fe-S dependent.

## DISCUSSION

The SlyD protein is a PPIase and multifunctional metallochaperone for which very few targets have been established *in vivo*. Given its functions, SlyD has the potential of displaying a wide targetome and to modulate numerous cellular functions by accelerating isomerization of peptidyl-proline bonds that are required for full enzymatic activity. However, SlyD targets are difficult to detect as no specific signature has been identified. Here, we tackled this question by applying a two-hybrid screen to search for novel SlyD interactors in *E. coli*, an easily tractable model organism, and in *H. pylori*, an important pathogen, and our study model. Many of the identified interactors contain metal cofactors in agreement with the metallochaperone function of SlyD. Several SlyD interactors of *E. coli* and *H. pylori* had similar or even identical functions strengthening our findings. In both *E. coli* and *H. pylori*, SlyD was shown to interact with Fe-S clusters’ biogenesis proteins and with some Fe-S-containing enzymes.

Fe-S clusters rank among the oldest and most conserved prosthetic groups found in proteins (33). *E. coli* is predicted to contain about 200 Fe-S proteins that constitute about 4% of the total proteome and are involved in a large variety of reactions and cellular processes, with one-third in respiration and the TCA cycle (38). Fe-S clusters are made and delivered to apo-proteins by dedicated machineries of different types, ISC and SUF for *E. coli* and MisSU for *H. pylori*. Here, we found that SlyD interacts in both *E. coli* and *H. pylori* with the cysteine desulfurases, *Ec*IscS, *Ec*SufS, and *Hp*MisS and with the scaffold, *Ec*IscU, *Hp*MisU, components of the Fe-S protein synthesis machineries, suggesting a possible metallochaperone function of SlyD in the activity of these machineries. Interestingly, previous *in vitro* studies have demonstrated that the function of IscU, one of the *E. coli* SlyD interactors, requires the *cis-trans* isomerization of two peptidyl-prolyl bonds in order to increase the efficiency of Fe-S formation and delivery (39). Since no PPIase has been assigned to this isomerization reaction, SlyD is an outstanding candidate to help the transition of the *E. coli* IscU metamorphic protein.

We applied several approaches to examine whether SlyD might play a role in the Fe-S biogenesis pathways in either *E. coli* or *H. pylori*. In *E. coli*, no defect of maturation of the IscR and NsrR Fe-S-dependent regulators was measured. In addition, the *E. coli* ∆*slyD*-∆*isc* and ∆*slyD*-∆*suf* double mutants were viable, indicating that the Fe-S cluster maturation of the two essential IspGH proteins is not completely blocked by SlyD inactivation. However, these results neither rule out the hypothesis that SlyD inactivation causes a moderate global decrease of the efficiency of Fe-S synthesis machineries nor that SlyD has an effect on specific proteins that we did not test. In contrast to the complexity of the two *E. coli* Fe-S machineries, the system of *H. pylori* only relying on MisSU is easier to study. In *H. pylori*, the ∆*slyD* mutant presents a striking decrease in the cellular content of both MisS and MisU proteins. That decrease is not caused by a diminished transcription of the *misSU* operon, and thus reflects a reduction in MisSU protein synthesis/stability. The effect is so strong that it is hardly conceivable that it has no effect on the activity of Fe-S proteins. Indeed, we found that the activity of the sole *H. pylori* Fe-S aconitase B is decreased by about 2.6-fold in a ∆*slyD* mutant. Aconitase B is an enzyme converting citrate to isocitrate and is part of the TCA cycle, which is of type VIII in *H. pylori* (see Fig. S3). Position of the Fe-S cluster in the aconitase structure renders this group particularly sensitive to oxidation, accordingly the cluster requires frequent repair. Aconitase activity is thus a good readout of the efficacity of the Fe-S biogenesis machineries.

These findings prompted us to search for phenotypes associated to the deletion of *slyD*. Most strikingly, we found that ∆*slyD* mutants in both *E. coli* and *H. pylori* are less susceptible to antibiotics. The two antibiotics tested act differently in distinct cellular compartments: ampicillin on the cell wall in the periplasm and gentamicin on translation on the ribosome. Our data with aconitase and the conserved two-hybrid interactions of SlyD with fumarase in both *E. coli* and *H. pylori* are suggestive of an impact of SlyD at the level of the TCA cycle. Indeed, in both bacteria, a ∆*fumC* deletion phenocopied the reduced antibiotic susceptibility of the *∆slyD* mutants. Data are accumulating in several bacteria linking a diminished metabolic activity, in particular that of the TCA cycle, with reduced susceptibility to antibiotics and increased persister formation (40). In particular, in *Staphylococcus aureus,* a mutant deficient in the sole fumarase FumC presents increased tolerance to diverse antibiotics, oxacillin (a β-lactam such as ampicillin), gentamicin, and ciprofloxacin (41). This tolerance has been associated with low ATP levels that could be confirmed by *in vivo* measurements (42). In the present work, we measured a lower cellular ATP content and reduced pmf in both the *H. pylori ∆slyD* and *∆fumC* mutants. This attests of a reduction in the cellular energetic status of these mutants that can be associated with diminished susceptibility to antibiotics.

Our data suggest a role of SlyD in Fe-S biogenesis. However, the FumC fumarase, a SlyD interactor conserved in *E. coli* and in *H. pylori*, does not contain a Fe-S cluster. To understand the basis of the phenotypic similarity of the *∆slyD* and *∆fumC* mutants, we decided to evaluate the activity of fumarase in *H. pylori* by measuring the intracellular concentrations of fumarate and malate. In contrast to *∆fumC*, the *∆slyD* mutant accumulated fumarate when malate was added to the culture. This suggests that fumarase activity itself is not significantly affected in the absence of SlyD. In contrast, malate was accumulated when either malate or fumarate was added to the culture suggesting that fumarase mainly functions in the fumarate to malate direction. These data also suggest that, in *H. pylori*, the activities of the enzymes before and after fumarase in the TCA cycle, namely malate quinone oxidoreductase (MQO) and fumarate reductase (FrdABC), both depending on Fe-S clusters for their functions, are affected in the ∆*slyD* mutant (see the *H. pylori* TCA cycle in Fig. S3). This could explain why the ∆*fumC* mutant, in which the TCA cycle is interrupted, phenocopies the ∆*slyD* mutant, deficient in Fe-S biogenesis. FrdB is an Fe_3_S_4_ enzyme and, in *H. pylori*, both MQO and FrdABC enzymes rely on menaquinone (MK) for their activities. Biosynthesis of MK relies on Fe-S SAM radical enzymes. Unfortunately, our measurements of total (oxidized and reduced) intracellular MK6 amounts in *H. pylori* WT compared to ∆*slyD* mutants did not reveal differences. However, we cannot exclude that, in an *H. pylori* ∆*slyD* mutant, MK6 is affected in its function and/or recycling as a consequence of the low MisSU protein amounts.

In favor of this hypothesis is the observation that overexpressing the Fe-S biogenesis proteins (MisSU) in an *H. pylori* ∆*slyD* mutant restores gentamicin susceptibility. The requirement on Isc-dependent Fe-S clusters allowing a pmf compatible with efficient gentamicin uptake has been demonstrated in *E. coli* (35). It is probable that the reduced pmf measured in the *H. pylori ∆slyD* mutant contributes to diminished gentamicin uptake and thus susceptibility. The lack of rescue of ampicillin susceptibility in the ∆*slyD* +p*misSU* strain suggests that higher MisSU amounts and/or other SlyD activities are needed to restore the activity of this antibiotic. In *E. coli*, only a small reduction in ampicillin susceptibility was observed in the IscS mutant (35).

Another phenotype of the *E. coli* and *H. pylori* ∆*slyD* mutants is, under microaerobic growth conditions, a reduced susceptibility to hypochlorite, a potent ROS producer generated by neutrophiles upon host colonization. Again, fumarase mutants phenocopy this resistance, both ∆*fumA* and ∆*fumC* in *E. coli* and ∆*fumC* in *H. pylori*. As discussed earlier, the *H. pylori ∆slyD* sensitivity to hypochlorite is restored in a ∆*slyD* mutant overexpressing *misSU* also indicating that this phenotype is most probably associated to a reduction in Fe-S clusters’ synthesis. Several interpretations to this phenotype can be proposed. The first one is that, under low oxygen conditions, the ∆*slyD* mutants, having a slowed-down TCA cycle and a reduced amount of Fe-S clusters, it becomes less sensitive to this oxidative stress. However, *H. pylori* is well equipped with ROS defense mechanisms, with a constitutive catalase and a superoxide dismutase (SOD). Alternatively, given that we previously showed that SlyD helps nickel entry in *H. pylori*, it might be that SlyD more generally affects metal uptake in both *E. coli* and *H. pylori* by acting on the transporters either directly or indirectly through the reduction of pmf. Several metals, in particular iron, react with ROS through the Fenton reaction to generate hydroxyl radicals, which are powerful oxidizing agents. Nevertheless, the precise mechanism associated to this phenotype requires more investigation.

In conclusion, we showed, for the first time, that a bacterial PPIase metallochaperone can act as a factor controlling central metabolic functions and susceptibility to oxidative stress and antibiotics. We previously characterized an *H. pylori* metallochaperone with comparable properties, the metal-binding GroES-homolog HspA, that acts as both a chaperone and a metal donor for [NiFe]-hydrogenase (43). Our data suggest that SlyD activity is required to optimize the Fe-S biogenesis of some enzymes particularly from the TCA cycle. SlyD is not essential for Fe-S cluster formation, but our data are compatible with a model in which this metallochaperone helps some proteins acquiring a functional conformation compatible with Fe-S transfer and/or help the cluster biogenesis process itself. SlyD seems to act only on a subcategory of Fe-S proteins. SlyD substrate specificity could correspond to a checkpoint in the distribution of Fe-S clusters, which might possibly be modulated by metabolic signals. We also showed that SlyD is required for antibiotic and oxidative stress resistance. While the flow of the TCA cycle is associated to antibiotic susceptibility, the mechanism underlying SlyD-mediated stress resistance is still to be explored further.

Finally, given the role of SlyD, understanding the role of this metallochaperone could help opening up new avenues to improve the antibiotic treatments to fight bacteria in refractory infections.

## MATERIALS AND METHODS

### Bacterial strains and growth conditions

The *H. pylori* strains used in this study are derivatives of B128, and the *E. coli* are derivatives of MG1655 (Table S3). *H. pylori* strains were grown at 37°C under microaerophilic conditions (6% O_2_, 14% CO_2_, 80% N_2_) on blood agar base 2 (Oxoid) plates supplemented with 10% defibrinated horse blood or Brucella broth agar (BD Difco) plates (designated BB) supplemented with 10% fetal calf serum (FCS, Eurobio). For liquid cultures, we used Brucella broth (BD Difco), supplemented with 10% fetal calf serum (Eurobio). For *H. pylori*, all plates and liquid cultures were supplemented with the following antibiotics-anti-fungal cocktail: amphotericin B 2.5 µg·mL^−1^, polymyxin B 0.31 µg·mL^−1^, trimethoprim 6.25 µg·mL^−1^ and vancomycin 12.5 µg·mL^−1^. Selection of *H. pylori* mutants and transformants was performed using kanamycin 20 µg·mL^−1^, chloramphenicol 6 µg·mL^−1^, streptomycin 10 µg·mL^−1^, or apramycin 10 µg·mL^−1^. *E. coli* strains were grown on solid or liquid Luria-Bertani (LB) medium. XL1-Blue was used for subcloning and as a host for the preparation of the plasmids employed to transform *H. pylori. E. coli* MG1655 strain was used for the construction of mutants and phenotypic analysis. *E. coli* strain BTH101 and DHM1 were used for BACTH (Tables S1 to S3) and plates were supplemented with X-Gal (5-bromo-4-chloro-3-indolyl-beta-D-galactopyranoside) at 40 µg·mL^−1^ for the library screening. LB medium was supplemented with chloramphenicol 30 µg·mL^−1^, ampicillin 100 µg·mL^−1^, or kanamycin 50 µg·mL^−1^ when required. When indicated, 0.1 mM of isopropyl β-D-1-thiogalactopyranoside (IPTG, EuroMedex) was added to the plates or cultures.

### Molecular techniques

Molecular biology experiments were performed according to standard procedures and the supplier (Fermentas) recommendations. NucleoBond Xtra Midi Kit (Macherey-Nagel) and QIAamp DNA Mini Kit (Qiagen) were used for plasmid preparations and *H. pylori* genomic DNA extractions, respectively. PCR was performed with either DreamTaq DNA polymerase (ThermoFisher), Q5 DNA polymerase (Biolabs), or PrimeSTAR Max DNA polymerase (Takara) when the product required high-fidelity polymerase.

### Construction of *E. coli* mutant strains

To obtain an *E. coli* MG1655 mutant carrying a complete deletion of the *slyD* gene (Table S3), we first replaced the entire *slyD* gene by an apramycin resistance cassette into strain CF10230 (44) using the primers shown in Table S4 and the *E. coli* recombination strategy of reference 45. Then, this mutation was transduced with phage P1vir into strain MG1655 and validated by PCR (Tables S3 and S4). The MG1655 ∆*slyD* mutant was complemented with plasmids derived from vector pILL2150 (46) expressing either the full-length *E. coli* SlyD protein (p*slyD*) or a SlyD protein truncated of its 42 last amino acids (p*slyD-∆Cter*) (Table S3). These plasmids were obtained by cloning of PCR products obtained with the primers shown in Table S4 between the *Bam*HI and *Eco*RI restrictions sites of vector pILL2150. The plasmids were validated by PCR and sequencing.

The MG1655 *E. coli ∆fumA* and *∆fumC* mutants were obtained by P1vir phage transduction of individual mutations of the Keio mutant collection and selection on kanamycin plates (47). The triple *∆fumABC* mutant was obtained by successive P1vir phage transductions of the individual *fum* mutations followed by elimination of the kanamycin cassette by the method proposed by reference (48). All the mutants were validated by PCR.

To test whether the SlyD protein impacted the Fe-S clusters’ formation in *E. coli*, two reporter fusions were used as we previously described (35). These fusions were expressed from plasmids P*_hmpA_-lacZ* and P*_iscR_-lacZ* that were transformed into MG1655 *iscAU::cat* and MG1655 ∆*suf::cat* mutants, respectively (34) (Table S3). Then, the *slyD* deletion was transduced from strain MG1655 ∆*slyD* by phage P1vir into strains Δ*iscAU::cat* P*_hmpA_-lacZ* and Δ*suf::cat* P*_iscR_-lacZ* (Table S3). These constructs were verified by PCR and sequencing.

### Construction of *H. pylori* mutant strains

The *H. pylori* B128 *∆slyD*, *slyD-∆Cter* mutants, and *∆slyD* mutant recomplemented by a WT *slyD* copy at the locus were previously described (11) (Table S3). Unmarked *oorD, fumC,* and *hemN* deletion mutants of *H. pylori* strain B128 (Table S3) were constructed by allelic exchange as previously described (21). We used a *H. pylori* suicide plasmid derived from pGEMT, in which about 500 bp of the 5′-end and the 3′-end regions immediately flanking the open reading frames of the genes to be deleted were cloned on each side of a *difH-cat-rpsL-difH* cassette (primers are listed in Table S4). These plasmids were used to naturally transform *H. pylori* strain B128 that we made streptomycin resistant. The insertion of the cassette by homologous recombination was selected on blood agar plates containing chloramphenicol 6 µg·mL^−1^. Removal of the cassette was achieved by plating the Cm^R^ clones on blood agar plates containing streptomycin 10 µg·mL^−1^. Unmarked deletions of the *oorD, fumC* and *hemN* gene were verified by PCR and sequencing of the gene regions.

The *E. coli-H. pylori* shuttle vector pILL2157 was used to construct a plasmid expressing the *misS-misU* operon under control of an inducible promoter (46). For this construct, the *misS-misU* operon was PCR amplified from B128 chromosomal DNA using the primers shown in Table S4, digested with *Spe*I and cloned into pILL2157. Depending on the orientation of the cloned PCR fragment, two plasmids were obtained (Table S3). In the first one, designated p*misSU*, the genes are under the control of the IPTG-inducible promoter. In the opposite orientation, the genes have no promoter and the plasmid designated pcontrol served as negative control. The two plasmids were introduced by natural transformation into the *H. pylori ∆slyD* mutant as reported previously (21) (Table S3). RT-qPCR was used to validate that, upon induction by IPTG, the *misS-misU* genes were indeed overexpressed (4–5 fold) in a *∆slyD H. pylori* strain containing the p*misS-misU* plasmid as compared to a *∆slyD* strain containing a control plasmid that does not express the *misS-misU* genes (pcontrol) (Fig. S1B).

*H. pylori* strains carrying on the chromosome, under control of their native promoter, *misS* FLAG-tag or *misU* FLAG-tag fusions were obtained as follows. Overlap extension PCR was used to construct a fragment comprising, in this order, 500 bp including the entire *misS* or *misU* genes fused to the FLAG-tag sequence at its 3′-extremity followed by a kanamycin resistance cassette and finally 500 bp corresponding to the downstream region of the *misS or misU* gene. The final PCR product was directly naturally transformed into *H. pylori* WT strain and *∆slyD* mutant and allelic exchange was selected on kanamycin as in reference 21. Correct insertion of the fusion and cassette were verified by PCR and sequencing.

### BACTH tests and β-galactosidase assays

The BACTH assay is based on the reconstitution of adenylate cyclase activity in a *cya^−^ E. coli* strain as a result of the interaction between two proteins: a bait and a prey fused to two separate domains (T18 and T25) of the *Bordetella pertussis* adenylate cyclase. The reconstitution of cyclase activity is measured by the activity of the β-galactosidase enzyme expressed from the *lacZ* reporter gene (25). Empty pNKT25 and pUT18C vectors served as controls in combination with every fusion construct shown in Fig. 1 and 2 (49). For every control combination, the reporter β-galactosidase activity is below 50 Miller units corresponding to nonsignificant background activity.

First, two genome-wide screens were performed in order to identify novel interactors of SlyD from *E. coli* and from *H. pylori*. The *E. coli slyD* gene was PCR amplified from strain MG1655 with primers shown in Table S4, cloned into vector pKT25 (Table S3) and introduced into strain BTH101. The *E. coli* library consisted of DNA fragments ranging from 300 to 1,000 bp from strain MG1655 cloned into vector pUT18C (Table S3). The construction of the library was described in detail in reference 50. *E. coli* pKT25 *slyD* strain was transformed with the library, transformants were selected at 30°C on LB agar plates containing kanamycin and ampicillin with the addition of X-Gal (5-bromo-4-chloro-3-indolyl-beta-D-galactopyranoside) at 40 µg·mL^−1^. Blue colonies were reisolated and the insert of the pUT18C plasmid was PCR amplified and sequenced (Table S4). The *H. pylori slyD* gene was PCR amplified from strain B128 with primers shown in Table S4, cloned into vector pNKT25 (Table S3), and introduced into strain BTH101. The same strategy was used for the *H. pylori slyD* screening against a DNA library prepared from *H. pylori* strain 26695 using the same strategy than the *E. coli* library (50, 51) and cloned into vector pUT18C (Table S3).

In a second time, several pairwise interactions were tested by BACTH with both *E. coli* SlyD and *H. pylori* SlyD proteins. For these assays, the genes of interest were PCR amplified with primers listed in Table S4 from chromosomal DNA of *E. coli* MG1655 or *H. pylori* B128 strain and cloned into vector pUT18 (Table S3). The two plasmids expressing fusions to be tested were co-transformed in *E. coli* strain BTH101 and transformants were selected on LB agar plates containing kanamycin and ampicillin at 30°C. Then, 5mL of LB medium supplemented with antibiotics and IPTG 10^−3^ M were inoculated with the transformant clones and incubated overnight at 30°C. Quantification of the interactions in strains carrying each plasmid combination was obtained by measurement of the β-galactosidase activity expressed in Miller units that was performed in at least three independent experiments and at least five replicates as in reference 49.

### Measurement of aconitase activity

#### Cell lysis

*H. pylori* cells were harvested by centrifugation and lysed after spheroplast preparation using the *E. coli* protocol (52). Cell pellets normalized to the same absorbance reading at 600 nm were suspended in 10 mM Tris-HCl (pH 7.5) containing 0.7 M sucrose and 1 mM phenylmethylsulfonyl fluoride. Lysozyme (0.2 mg·mL^−1^) and EDTA (10 mM) were added, and the suspensions were incubated for 20 min at 4°C. The samples were centrifuged for 5 min at 10,000 rpm in an Eppendorf microcentrifuge and the supernatants were removed. The pellets were freeze-thawed, resuspended in cold water, and then sonicated for 10 s with a Branson digital sonifier.

#### Aconitase assay

The aconitase activity of cell lysates was measured spectrophotometrically by monitoring the formation of *cis*-aconitate from isocitrate at OD 240 nm using a Jasco V-730 spectrophotometer. Samples were added to 3 mL of 50 mM Tris-HCl (pH 7.4) containing 20 mM isocitrate and 0.5 mM MnCl_2_ to initiate the reaction at 30°C (53). Specific activities were calculated using the molar extinction coefficient of 3.6 mM^−1^·cm^−1^ for *cis*-aconitate and the protein content determined by the Bradford assay.

### Western blotting

Western blots were performed with 20 µg of proteins loaded and separated on a 4–20% Mini-Protean TGX Stain-Free precast protein gel (BioRad) and subsequently electro-transferred on a polyvinylidene difluoride (PVDF) membrane (Biorad) by TransBlot Turbo system (Biorad). Fractionation to separate soluble fraction from membrane fraction was performed as previously established for *H. pylori* cells and is described in detail in reference 11. The *H. pylori* MisS-FLAG and MisU-FLAG proteins were detected with anti-FLAG M2 antibodies (dilution 1/1,500) produced in mice (F3165, Sigma). HRP-Anti-mice was used as secondary antibodies at 1:10,000 dilution and the detection was achieved with the ECL reagent (Thermo Fisher). A stain-free gel was used to be able to precisely normalize the amounts of proteins in each lane of the gel used for the western (see Fig. S1A).

### RT-qPCR

Three independent *H. pylori* liquid cultures of 30 mL each were grown for 16 h until OD 1, centrifuged for 15 min at 4000 *g,* treated with RNA protect solution (Qiagen), and stored at −80°C. Then, cells were lysed, and RNA was extracted with the “Total RNA Purification Plus Kit” (Norgen Biotek 48300). DNA was removed from RNA preparations by incubation for 30 min at 37°C with 2 U/µL of Turbo RNase-free DNase (Invitrogen). Synthesis of cDNA was carried out following the manufacturer’s protocol using SuperScript IV First-Strand Synthesis System (ThermoFisher), starting with 1 µg total RNA. cDNA was diluted to 10 ng/µL in nuclease-free water. Finally, RNA transcripts were quantified on an Applied Biosystems StepOnePlus PCR machine using the Power SYBR Green PCR Master Mix (Applied Biosystems), 900 nM of each primer (Table S4), and 30 ng of total cDNA. PCR products were amplified and detected with an Applied Biosystem (Thermofisher) instrument. The cycling conditions were as follows: one cycle at 95°C for 10 min, 45 cycles at 95°C for 15 s and 60°C for 2 min, and 80 cycles at 55°C for 30 s with a 0.5°C increase every 30 s. The transcript levels were normalized to the level of the housekeeping *ppK* gene (encoding polyphosphate kinase, *hp1010*) as previously validated (54). The data correspond to at least three independent experiments with two technical replicates each time.

### Measurement of the susceptibility of *E. coli* and of *H. pylori* to antibiotics and oxidative stress

In order to test whether the absence of the SlyD protein might impact the susceptibility of *E. coli* and *H. pylori* to antibiotics, we first determined, under our liquid growth conditions, the MIC50 (the MIC value at which 50% of the cells have lost viability) for ampicillin and gentamicin for both WT *E. coli* and *H. pylori* strains. *E. coli* MG1655 strains were grown in liquid LB medium, *H. pylori* B128 strains in liquid *Brucella* broth. Under our test conditions, MIC50 for gentamicin, was 3 µg·mL^−1^ and 1.5 µg·mL^−1^ for *H. pylori* and *E. coli*, respectively. For ampicillin, MIC50 was 7.5 µg·mL^−1^ for both *H. pylori* and *E. coli*. For our tests, we chose sublethal concentrations that were slightly above the MIC50 for both *H. pylori* and *E. coli*: 5 µg·mL^−1^ for gentamicin and 10 µg·mL^−1^ for ampicillin.

The experiment started with an overnight preculture without added antibiotics, the preculture was diluted to an OD of 0.2 and was further grown during 2 h. When the strain contained a plasmid, chloramphenicol at 20 µg·mL^−1^ and IPTG at 10 µM were added to the cultures. The cultures were incubated at 37°C under agitation at 150 rpm, aerobically for *E. coli* or under microaerophilic conditions for *H. pylori*. Then, the cultures were divided in two, one without antibiotics and one to which the antibiotic was added at the indicated concentrations. The time of exposure to antibiotics was chosen in order to obtain a reduction of relative viability (ratio of cell viability with antibiotics versus without antibiotics in %) of at least 50% for the WT strains. These times of exposure to antibiotics were 60 min for the experiment with ampicillin and 30 min with gentamicin, times at which aliquots were taken from each culture. From these aliquots, the number of viable bacteria was measured by bacterial numeration after plating of serial dilutions to 10^−6^ on LB or *Brucella* agar medium for *E. coli* and for *H. pylori*, respectively, providing CFUs (colony forming units). The Y axis of Fig. 4 and 5 presents the “Relative cell viability with antibiotics versus without antibiotics expressed as a percentage.”

For the tests of susceptibility to hypochlorite, the procedure was similar. First, the conditions at which a reduction of relative cell viability (ratio of cell viability with hypochlorite versus without hypochlorite in %) of at least 50% for the *E. coli* and *H. pylori* WT strains was established using different concentrations of hypochlorite and exposure times. The chosen conditions were overnight incubation under microaerophilic conditions and exposure to the oxidative stress generated by hypochlorite at 1.5 µM for *E. coli* and 1 mM for *H. pylori*. Aliquots were taken and the number of viable bacteria was measured by bacterial numeration after plating of serial dilutions to 10^−6^ on LB or Brucella agar medium for *E. coli* and for *H. pylori*, respectively, providing CFUs. The Y axis of Fig. 6 presents the “Relative cell viability with hypochlorite versus without hypochlorite expressed as a percentage”.

### ATP extraction and assay

*H. pylori* WT, *∆slyD,* and *∆fumC* strains were exponentially grown in 30 mL of liquid medium until OD 1. Then, they were harvested by centrifugation at room temperature for 4 min at 5,000 *g*. Metabolites from the resulting cell pellets were extracted immediately using 300 µL of a solvent mixture of acetonitrile/methanol/H_2_O (40/40/20) for 15 min at 4°C. Mixtures were subsequently spun in a microfuge for 5 min at maximum speed and 4°C to separate insoluble materials from the extracted metabolites. The resulting pellets were then re-extracted twice with 200 µL of solvent at 4°C. The supernatants were pooled to yield 700 µL of final extract. Metabolites were lyophilized and subsequently diluted in water for ATP assays. ATP content was determined by a luciferase-based ATP bioluminescence assay kit (BacTiter-Glo Microbial cell viability assay, Promega). Luminescence values were determined using a 10 s RLU signal integration time and measured using a Centro XS^3^ LB960 Luminometer (Berthold Technologies). ATP concentrations were calculated based on values determined using serial dilutions of known amounts of ATP and expressed as a function of the OD_600_ of the corresponding culture. Three independent experiments were performed in triplicates.

### Measurement of *H. pylori* PMF by flow cytometry

*H. pylori* WT, *∆slyD,* and *∆fumC* strains were inoculated at OD 0.025 and grown in liquid medium in triplicate for 16 h. For the measurement of pmf, samples of 10^7^ live cells per experimental condition were taken, washed, and stained with 25 nM MitoTracker Red CMXRos (Invitrogen), a PMF-sensitive dye. As a control for PMF depletion, the bacteria were treated with 500 µM of the protonophore TCS (3,3′,4′,5-tetrachlorosalicylanilide; Fisher Scientific) as in reference 37. The fluorescent signal from 50,000 individual bacteria per condition was measured by flow cytometry with a MCSQuant VYB analyzer (Miltenyi Biotec) (Y2 channel, λ_ex_=561 nm and λ_em_=605–625 nm) after calibration. The experiment was performed three times. Data were analyzed with FlowJo V10.

### Intracellular concentrations of fumarate and malate in *H. pylori* strain

To measure the concentrations of malate and fumarate in *H. pylori* cells, we used the MAK067 Malate and MAK060 Fumarate Assay Kits (Sigma Aldrich). In these assays, malate is specifically oxidized to generate a product, which reacts with a substrate probe to generate color (λmax = 450 nm). The fumarate assay also results in a colorimetric product (450 nm) proportional to the fumarate. For these assays, 30 mL of *H. pylori* cultures were prepared by liquid medium inoculation at initial OD 0.025 and growth until OD 1. *H. pylori* cells were collected by centrifugation, concentrated in water to OD 50, and further lysed by sonification. The cell debris was eliminated by centrifugation and the supernatant was boiled during 10 min to denaturate the proteins. The test was first validated with *H. pylori* cell extracts to which a defined range of malate or fumarate was added. The concentration of malate and fumarate was calculated from the standard curve and expressed as nmol of malate or fumarate per OD cell culture. Malate and fumarate concentrations of WT, *∆slyD,* and *∆fumC* cells were assayed without any addition or upon the addition to the culture medium of 5 mM malate or 25 mM fumarate. Several concentrations were first tested (Fig. S4), *H. pylori* growth was found to be strongly affected by the addition of 15 mM malate. In the presence of 10 mM malate, only growth of the *∆slyD* and *∆fumC* mutants were strongly reduced with a most prominent effect in the absence of fumarase. All the strains grew similarly with 5 mM malate. In contrast, fumarate was nontoxic until the concentration of 50 mM similarly to what is observed in other bacteria such as *E. coli*. These experiments were performed three times in triplicate.

### Statistical analysis

The Student *t*-test was used to determine significant differences of the means of the data for all the experiments except for the aconitase assay, for which the Wilcoxon test was applied. Error bars represent the standard deviation, with * (*P* < 0.05), ** (*P* < 0.01), *** (*P* < 0.001), **** (*P* < 0.0001) indicating that the mean values are significantly different and ns that they are not significantly different (*P* > 0.05).

## Data Availability

All data needed to evaluate the conclusions in the paper are present in the paper and/or the supplemental material. Additional data related to this paper may be requested from the authors.

## SUPPLEMENTAL MATERIAL

Figure S1 mbio.00967-23-s0001.docx Western blot of MisSU and RTqPCR of *misSU*.

Figure S2 mbio.00967-23-s0002.pdf Representative flow cytometry histograms of different *H. pylori* cells.

Figure S3 mbio.00967-23-s0003.pdf Representation of the TCA cycle of *H. pylori*.

Figure S4 mbio.00967-23-s0004.docx Growth of *H. pylori* strains in the presence of malate or fumarate.

Table S1 mbio.00967-23-s0005.docx Results of the BACTH screen of the *Escherichia coli Ec*SlyD interactors.

Table S2 mbio.00967-23-s0006.docx Result of the BACTH screen of the *H. pylori Hp*SlyD interactors.

Table S3 mbio.00967-23-s0007.docx List of strains and plasmids used in the study.

Table S4 mbio.00967-23-s0008.docx List of primers used in the study.

ASM does not own the copyrights to Supplemental Material that may be linked to, or accessed through, an article. The authors have granted ASM a non-exclusive, world-wide license to publish the Supplemental Material files. Please contact the corresponding author directly for reuse.
