# Supplementary material for: The SlyD metallochaperone targets iron-sulfur biogenesis pathways and the TCA cycle
Source: mBio. 2023 Aug 16;14(5):e00967-23. doi: 10.1128/mbio.00967-23 (PMC10653786; doi:10.1128/mbio.00967-23)
Supplement: Figure S1 — Western blot of MisSU and RTqPCR of misSU. [file mbio.00967-23-s0001.docx]

**Supp Figure S1:**

**A)** Stain free gel of the western blot shown in Figure 3, that was used to normalize the amounts of proteins in each lane.

**B)** RT-qPCR measurement of the expression of the *misS* and *misU* genes in the *H. pylori* WT strain, in the *∆slyD* strain with the pcontrol plasmid and the ∆*slyD* mutant carrying the p*misS-misU* plasmid in the presence of IPTG, expressed as a relative value of the WT. These results are the means with the standard deviations of four independent experiments.
