# Supplementary material for: The SlyD metallochaperone targets iron-sulfur biogenesis pathways and the TCA cycle
Source: mBio. 2023 Aug 16;14(5):e00967-23. doi: 10.1128/mbio.00967-23 (PMC10653786; doi:10.1128/mbio.00967-23)

Supp Figure S2: Representative flow cytometry histograms of different *H. pylori* cells stained with a PMF-sensitive dye, MitoTracker Red CMXROS dye. Histograms of mean fluorescence intensity (A.U, arbitrary units) of cells from strains WT, Δ*fumC* and Δ*slyD* stained with the PMF-sensitive dye. Wild type cells treated with TCS served as a control for PMF dissipation conditions. For each condition, 50,000 cells were analyzed.

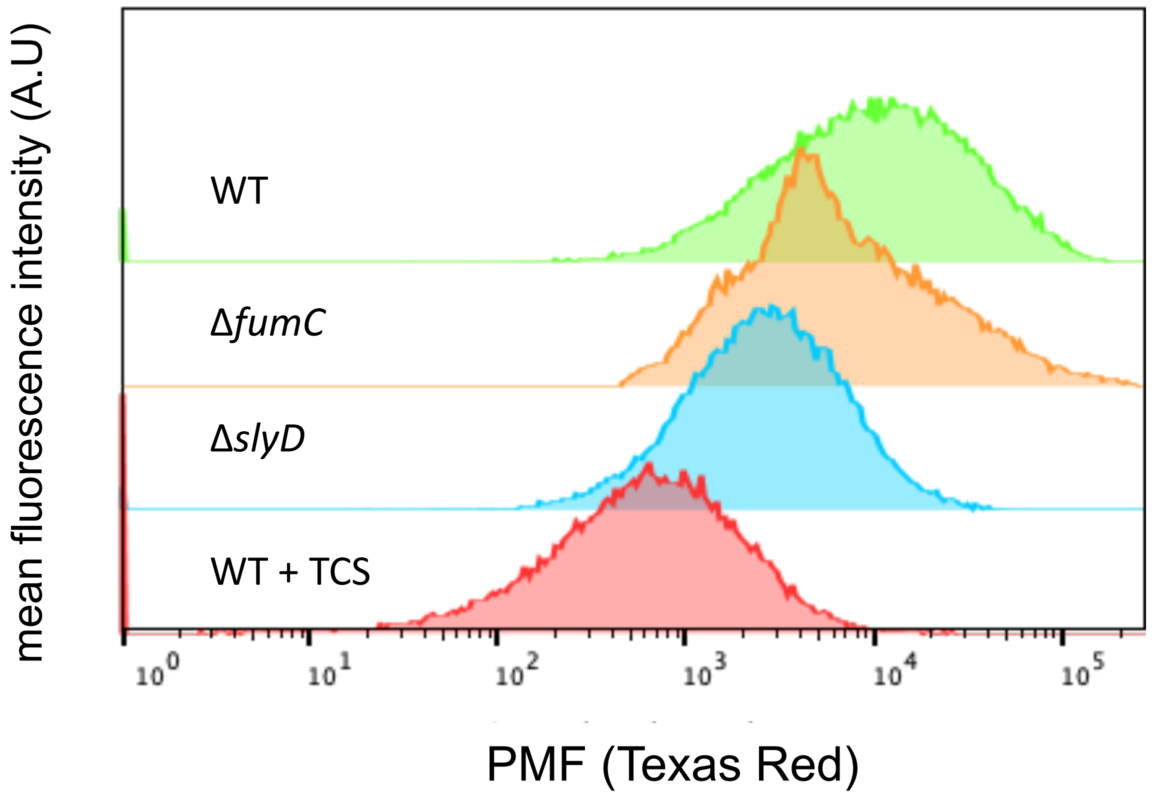

Supplement: Figure S2 — Representative flow cytometry histograms of different H. pylori cells. [file mbio.00967-23-s0002.pdf]
