# Supplementary figures and images for: The SlyD metallochaperone targets iron-sulfur biogenesis pathways and the TCA cycle
Source: mBio. 2023 Aug 16;14(5):e00967-23. doi: 10.1128/mbio.00967-23 (PMC10653786; doi:10.1128/mbio.00967-23)

**Supp Figure S3:** Representation of the TCA cycle of *H. pylori* (Type VIII cycle)

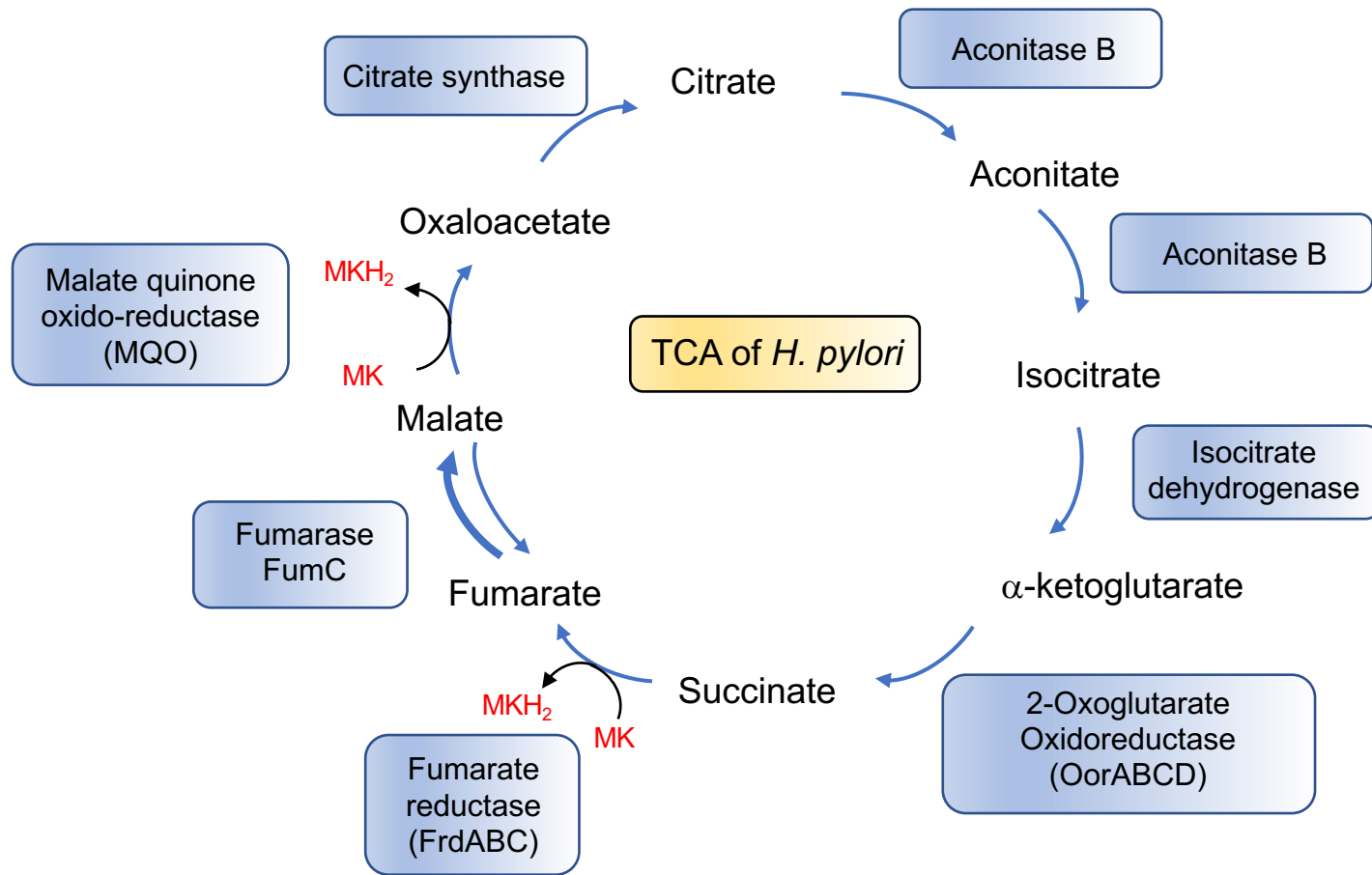

Supplement: Figure S3 — Representation of the TCA cycle of H. pylori. [file mbio.00967-23-s0003.pdf]
