# Supplementary material for: The SlyD metallochaperone targets iron-sulfur biogenesis pathways and the TCA cycle
Source: mBio. 2023 Aug 16;14(5):e00967-23. doi: 10.1128/mbio.00967-23 (PMC10653786; doi:10.1128/mbio.00967-23)

**Supp Figure S4:** Growth of *H. pylori* WT, ∆*slyD* and ∆*fumC* strains in the presence of malate or fumarate at the indicated concentrations. The Y axis corresponds to the percentage of relative OD with fumarate or malate versus conditions without any addition. These results are the means with the standard deviations of three independent experiments.


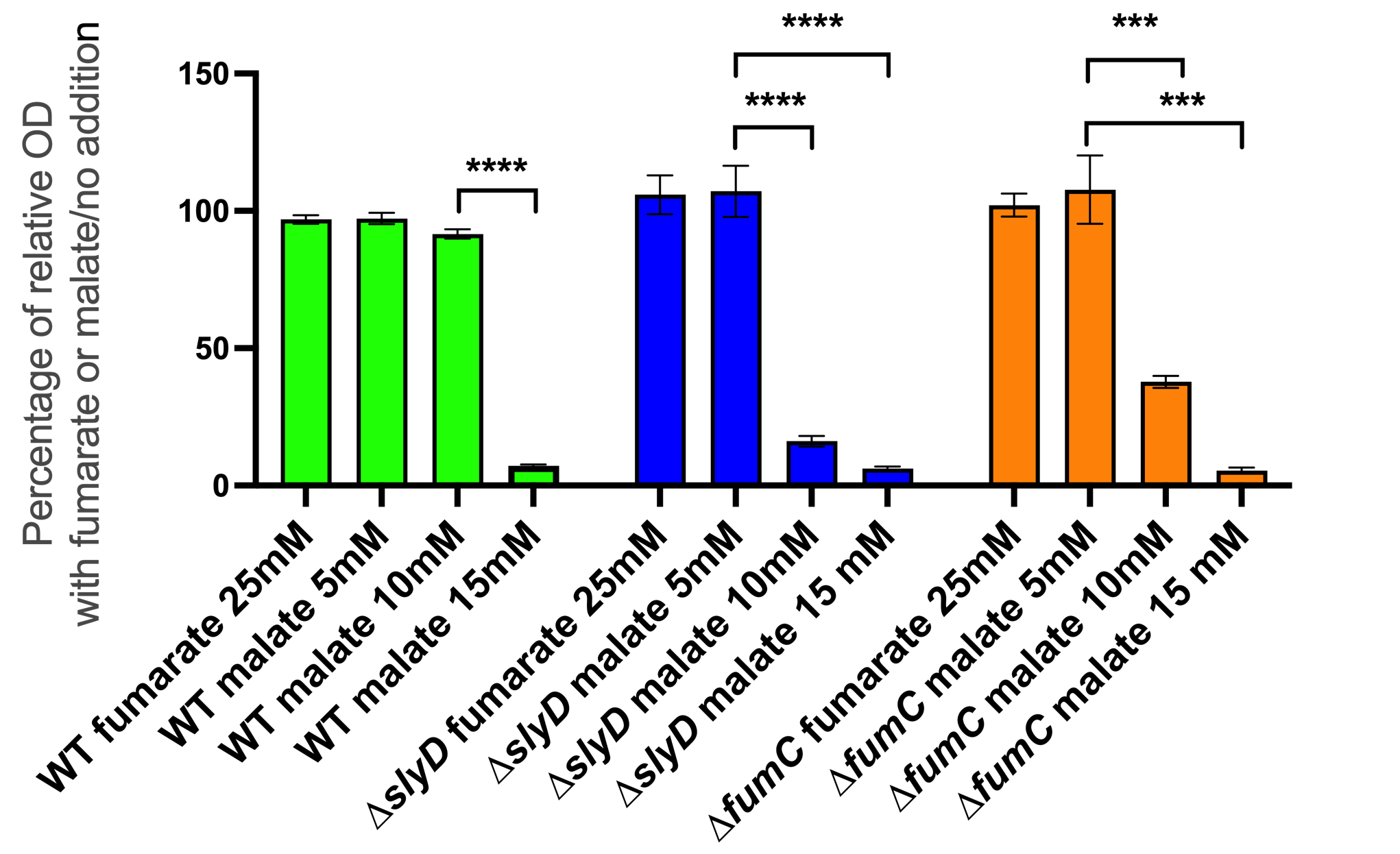

Supplement: Figure S4 — Growth of H. pylori strains in the presence of malate or fumarate. [file mbio.00967-23-s0004.docx]
