# Supplementary material for: The SlyD metallochaperone targets iron-sulfur biogenesis pathways and the TCA cycle
Source: mBio. 2023 Aug 16;14(5):e00967-23. doi: 10.1128/mbio.00967-23 (PMC10653786; doi:10.1128/mbio.00967-23)
Supplement: Table S1 — Results of the BACTH screen of the Escherichia coli EcSlyD interactors. [file mbio.00967-23-s0005.docx]

## Supplementary table S1: Result of the BACTH screen of the *Escherichia coli Ec*SlyD interactors, only clones that had in frame fusions are shown.

| Gene | Function of the protein |
| --- | --- |
| *wzyE* | Putative ECA polymerization protein |
| *fumA* | Class I aerobic fumarate hydratase A (fumarase A) (Fe_4_S_4_ cluster) |
| *yfjH* | CP4-57 prophage |
| *mdtM* | multidrug efflux system protein |
| *yjiP* | Pseudogene, transposase_31 family protein |
