# Supplementary material for: The SlyD metallochaperone targets iron-sulfur biogenesis pathways and the TCA cycle
Source: mBio. 2023 Aug 16;14(5):e00967-23. doi: 10.1128/mbio.00967-23 (PMC10653786; doi:10.1128/mbio.00967-23)
Supplement: Table S2 — Result of the BACTH screen of the H. pylori HpSlyD interactors. [file mbio.00967-23-s0006.docx]

## Supplementary table S2: Result of the BACTH screen of the *Helicobacter pylori Hp*SlyD interactors, only clones that had in frame fusions are shown.

| Gene | ORF number in *H. pylori* B8 (B128) | Function of the protein |
| --- | --- | --- |
| *hemN* | *HPB8_RS04115* | Coproporphyrinogen oxidase III(Fe_4_S_4_ cluster) |
| *oorD* | *HPB8_RS03740* | 2-oxoglutarate-acceptor oxidoreductase subunit D(Fe_4_S_4_ cluster) |
| *trpB* | *HPB8_RS00955* | Tryptophan synthase beta subunit |
| *hdpA or csd3* | *HPB8_690* | HdpA membrane protein with both D,D-carboxypeptidase and D,D-endopeptidase activities  (Zinc binding site) |
| *fumC** | *HPB8_RS00725* | Fumarate hydratase C (fumarase C) |
| *groEL* | *HPB8_1615* | Molecular chaperone GroEL(Mg binding site) |
| *dnaJ* | *HPB8_1142* | Chaperone protein DnaJ(Zinc binding site) |
| def | *HPB8_RS04740* | Peptide deformylase(Fe binding site) |
| *homA* | *Ortholog of hp0710* | Outer membrane protein, HomA |

*: two independent clones were obtained
