# Supplementary material for: The SlyD metallochaperone targets iron-sulfur biogenesis pathways and the TCA cycle
Source: mBio. 2023 Aug 16;14(5):e00967-23. doi: 10.1128/mbio.00967-23 (PMC10653786; doi:10.1128/mbio.00967-23)
Supplement: Table S3 — List of strains and plasmids used in the study. [file mbio.00967-23-s0007.docx]

## Supplementary table S3: list of the strains and plasmids used in this study.

## Strains used in this study

| **Strains** | **Relevant characteristics** | **Reference** |
| --- | --- | --- |
| *Escherichia coli* | | |
| MG1655 | F-, lambda, *ilvG*, *rfb*-50, *rph*-1, ∆*lac* | (1) |
| BTH101 | F-, *cya-99, araD139, galE15, galK16, rpsL1 (*Str^R^*), hsdR2, mcrA1, mcrB1* | (2) |
| DHM1 | F-, *cya-854, recA1, endA1, gyrA96* (Nal^R^), *thi1, hsdR17, spoT1, rfbD1, glnV44*(AS) | (2) |
| XL1-Blue | *recA1*, *endA1*, *gyrA96*, *thi-1*, *hsdR17*, *supE44 relA1 lac* [F*´ proAB, lacI^q^Z*, *ΔM15*, Tn10 (TetR)] | Agilent Technologies |
| MG1655 Δ*slyD* | F- lambda, *ilvG*, *rfb*-50, *rph*-1, ∆*lac*, Δ*slyD::apra* | This work |
| MG1655 Δ*slyD* + p*slyD* | F- lambda, *ilvG*, *rfb*-50, *rph*-1, ∆*lac*, Δ*slyD::apra,* pILL2150 with *slyD* | This work |
| MG1655 Δ*slyD* + p*slyD-∆Cter* | F- lambda, *ilvG*, *rfb*-50, *rph*-1, ∆*lac*, Δ*slyD::apra,* pILL2150 with *slyD-∆Cter* | This work |
| MG1655 ∆*fumA* | Unmarked *fumA* deletion mutant | This work |
| MG1655 ∆*fumC* | Unmarked *fumC* deletion mutant | This work |
| MG1655 ∆*fumABC* | Unmarked *fumA-B-C* deletion mutant | This work |
| DV1564 | MG1655 Δ*iscAU::cat* + P*_hmpA_-lacZ* | (4) |
| DV1475 | MG1655 Δ*iscAU::cat* + P*_iscR_-lacZ* | (4) |
| DV1306 | MG1655 Δ*suf::cat* + P*_hmpA_-lacZ* | (4) |
| DV1473 | MG1655 Δ*suf::cat* + P*_iscR_-lacZ* | (4) |
| DV1564 *∆slyD* | MG1655 Δ*slyD* Δ*iscAU::cat* + P*_hmpA_-lacZ* | This work |
| DV1475 *∆slyD* | MG1655 Δ*slyD* Δ*iscAU::cat* + P*_iscR_-lacZ* | This work |
| DV1306 *∆slyD* | MG1655 Δ*slyD* Δ*suf::cat* + P*_hmpA_-lacZ* | This work |
| DV1473 *∆slyD* | MG1655 Δ*slyD* Δ*suf::cat* + P*_iscR_-lacZ* | This work |
|  |  |  |
| *Helicobacter pylori* | | |
| 26695 | Reference wild type strain | (5) |
| B128 | Reference wild type strain | (6, 7) |
| B128 ∆*slyD* | Unmarked ∆*slyD* deletion mutant, Strepto^R^ | (3) |
| B128+pControl | Wild type strain with pControl, Cm^R^ | This work |
| B128 ∆*slyD+*pControl | Unmarked ∆*slyD* deletion mutant, with pControl, Strepto^R^, Cm^R^ | This work |
| B128 ∆*slyD*+p*misSU* | Unmarked ∆*slyD* deletion mutant, Strepto^R^ with p*misSU*, Strepto^R^, Cm^R^ | This work |
| B128 ∆*fumC* | Unmarked ∆*fumC* deletion mutant, Strepto^R^ | This work |
| B128 ∆*oorD* | Unmarked ∆*oorD* deletion mutant, Strepto^R^ | This work |
| B128 ∆*hemN* | Unmarked ∆*hemN* deletion mutant, Strepto^R^ | This work |
| B128 *slyD-ΔCter* | Unmarked *slyD ΔCter* deletion mutant, Strepto^R^ | This work |
| B128 ∆*slyD c-slyD* | Unmarked *slyD* deletion mutant, Strepto^R^, wild type *slyD* gene reintroduced at the chromosomal locus, Apra^R^ | (3) |
| B128 *misS*-FLAG | *misS* fused to a FLAG tag, Km^R^, Strepto^R^ | This work |
| B128 *misU*-FLAG | *misU* fused to a FLAG tag, Km^R^, Strepto^R^ | This work |
| B128 ∆*slyD misS*-FLAG | ∆*slyD*, *misS* fused to a FLAG tag, Km^R^, Strepto^R^ | This work |
| B128 ∆*slyD misU*-FLAG | ∆*slyD*, *misU* fused to a FLAG tag, Km^R^, Strepto^R^ | This work |

## Plasmids used in this study.

| **Vectors** | **Relevant characteristics** | **Reference** |
| --- | --- | --- |
| pKT25 | BACTH vector designed to express a protein fused in frame at its N-terminus with T25 domain of CyaA, p15 ori, Km^R^ | (2) |
| pKNT25 | BACTH vector designed to express a protein fused in frame at its C-terminus with T25 domain of CyaA, p15 ori, Km^R^ | (2) |
| pUT18 | BACTH vector designed to express a protein fused in frame at its C-terminus with T18 domain of CyaA, ColE1 ori, Amp^R^ | (2) |
| pUT18C | BACTH vector designed to express a protein fused in frame at its N-terminus with T18 domain of CyaA, ColE1 ori, Amp^R^ | (2) |
| pILL2157 | *E. coli*-*H. pylori* shuttle cloning vector, Cm^R^ | (8) |
| pILL2150 | *E. coli*-*H. pylori* shuttle derived cloning vector, Cm^R^ | (8) |
|  |  |  |
| **Plasmids** | **Relevant characteristics** | **Reference** |
| pKT25(*Ec-slyD*) | BACTH plasmid expressing the *Ec-*SlyD protein, Km^R^ | This work |
| pNKT25(*Hp-slyD*) | BACTH plasmid expressing the *Hp-*SlyD protein, Km^R^ | This work |
| pUT18(*Ec-iscA*) | BACTH plasmid expressing the *Ec-*IscA protein, Amp^R^ | Gift of L. Loiseau |
| pUT18(*Ec-iscU*) | BACTH plasmid expressing the *Ec-*IscU protein, Amp^R^ | Gift of L. Loiseau |
| pUT18(*Ec-iscS*) | BACTH plasmid expressing the *Ec-*IscS protein, Amp^R^ | Gift of L. Loiseau |
| pUT18(*Ec-sufS*) | BACTH plasmid expressing the *Ec-*SufS, Amp^R^ protein | Gift of L. Loiseau |
| pUT18C(*Ec-fumA*) | BACTH plasmid expressing the *Ec-*FumA protein, Amp^R^ | This work |
| pUT18C(*Ec-fumB*) | BACTH plasmid expressing the *Ec-*FumB protein, Amp^R^ | This work |
| pUT18C(*Ec-fumC*) | BACTH plasmid expressing the *Ec-*FumC protein, Amp^R^ | This work |
| pUT18(*Hp-misS*) | BACTH plasmid expressing the *Hp-*MisS protein, Amp^R^ | This work |
| pUT18(*Hp-misU*) | BACTH plasmid expressing the *Hp-*MisU protein, Amp^R^ | This work |
| pUT18C(*Hp-oorD*) | BACTH plasmid expressing the *Hp-*OorD protein, Amp^R^ | This work |
| pUT18C(*Hp-hemN*) | BACTH plasmid expressing the *Hp*-HemN protein, Amp^R^ | This work |
| pUT18C(*Hp-fumC*) | BACTH plasmid expressing the *Hp*-FumC protein, Amp^R^ | This work |
| pControl | Control pILL2157-derivative plasmid, Cm^R^ | This work |
| p*misSU* | pILL2157 derivative expressing *misSU*, Cm^R^ | This work |
| p*slyD* | pILL2150 derivative expressing *slyD*, Cm^R^ | This work |
| p*slyD-∆Cter* | pILL2150 derivative expressing *slyD-∆Cter*, Cm^R^ | This work |

**References**

1. Blattner F, Plunkett G 3rd, Bloch CA, Perna NT, Burland V, Riley M, Collado-Vides J, Glasner JD, Rode CK, Mayhew George F, Gregor Jason, Davis Nelson Wayne, Kirkpatrick Heather A, Goeden Michael A, Rose Debra J, Mau Bob, Shao Ying. 1997. The Complete Genome Sequence of *Escherichia coli* K-12. *Science* 277:1453–1462.
2. Karimova G, Ullmann A, Ladant D. 2000. A bacterial two-hybrid system that exploits a cAMP signaling cascade in *Escherichia coli. Methods Enzymol* 328:59–73.
3. Denic M, Turlin E, Michel V, Fischer F, Khorasani-Motlagh M, Zamble D, Vinella D, de Reuse H. 2021. A novel mode of control of nickel uptake by a multifunctional metallochaperone. *PLoS Pathog* 17:e1009193–e1009193.
4. Vinella D, Loiseau L, de Choudens SO, Fontecave M, Barras F. 2013. *In vivo* [Fe-S] cluster acquisition by IscR and NsrR, two stress regulators in *Escherichia coli. Mol Microbiol* 87:493–508.
5. Tomb J-F, White O, Kerlavage AR, Clayton RA, Sutton GG, Fleischmann RD, Ketchum KA, Klenk HP, Gill S, Dougherty BA, Nelson K, Quackenbush J, Zhou L, Kirkness EF, Peterson S, Loftus B, Richardson D, Dodson R, Khalak HG, Globek A, McKenney K, Fitzegerald LM, Lee N, Adams MD, Hickey EK, Berg DE, Gocayne JD, Utterback TR, Peterson JD, Kelley JM, Cotton MD, Weidman JM, Fuji C, Bowman C, Watthey L, Wallin E, Hayes WS, Borodovsky M, Karp PD, Smith HO, Fraser CM, Venter JC. 1997. The complete genome sequence of the gastric pathogen *Helicobacter pylori. Nature* 388:539–547.
6. McClain MS, Shaffer CL, Israel DA, Peek RMJ, Cover TL. 2009. Genome sequence analysis of *Helicobacter pylori* strains associated with gastric ulceration and gastric cancer. *BMC Genom* 10:3:doi: 10.1186/1471-2164-10-3.
7. Farnbacher M, Jahns T, Willrodt D, Daniel R, Haas R, Goesmann A, Kurtz S, Rieder G. 2010. Sequencing, annotation and comparative genome analysis of the gerbil-adapted *Helicobacter pylori* strain B8. *BMC Genom* 11:335.:doi: 10.1186/1471-2164-11-335.

8. Boneca IG, Ecobichon C, Chaput C, Mathieu A, Guadagnini S, Prevost M-C, Colland F, Labigne A, de Reuse H. 2008. Development of inducible systems to engineer conditional mutants of essential genes of *Helicobacter pylori. Appl Environ Microbiol* 74:2095–2102.
