# Supplementary material for: The SlyD metallochaperone targets iron-sulfur biogenesis pathways and the TCA cycle
Source: mBio. 2023 Aug 16;14(5):e00967-23. doi: 10.1128/mbio.00967-23 (PMC10653786; doi:10.1128/mbio.00967-23)
Supplement: Table S4 — List of primers used in the study. [file mbio.00967-23-s0008.docx]

**Supplementary table S4: primers used in this study**

| **Primer** | **Sequence of the primer** | **Restriction site** |
| --- | --- | --- |
| **Construction of the *H. pylori ∆slyD*, *fumC, oorD* and *hemN* unmarked mutants** | | |
| *∆slyD-flankA1* | GCCTGTGCTTTTGTGGCATACG |  |
| *∆slyD-flankA2* | CTGCACTTTTCAAACTTTTAAATCTCGAGTTATGAAGCTAAAATTTCTTCTTC | *XhoI* |
| *∆slyD-flankB1* | AAAGGATCCTAAGGTATAGGAGTCTTTAA | *Bam*HI |
| *∆slyD-flankB2* | GCAAGAATAAACCCCCTAG |  |
| *fumC-flankA1* | ATATCCACCCTCACCTGCCA |  |
| *fumC -flankA2* | GCCTCGAGACTAATCCTTTGAAA | *Xho*I |
| *fumC -flankB1* | CGGGATCCAACCTTGGGCGCTCGCATGA | *Bam*HI |
| *fumC -flankB2* | GGGGCTGAGCTTCTTGCTGTC |  |
| *oorD -flankA1* | GAGCGTGATTTTTAACCGCTTG |  |
| *oorD -flankA2* | AATCTCGAGTCATTCTCCTTTGTGCTAA | *Xho*I |
| *oorD -flankB1* | ATGGATCCGAGCTTGCGTGAGATTATTTCTGATG | *Bam*HI |
| *oorD -flankB2* | TCATCCATGAGCAAGAATACAGG |  |
| *hemN -flankA1* | ATATCCACCCTCACCTGCCA |  |
| *hemN -flankA2* | GCCTCGAGACTAATCCTTTGAAA | *Xho*I |
| *hemN -flankB1* | CGGGATCCAACCTTGGGCGCTCGCATGA | *Bam*HI |
| *hemN -flankB2* | GGGGCTGAGCTTCTTGCTGTC |  |
| *difHrpsLcat-1* | CCGCTCGAGATTTAAAAGTTTGAAAAG | *Xho*I |
| *difHrpsLcat-2* | CGCGGATCCATCGATCATTTAGTTATG | *Bam*HI |
| **Construction of *the E. coli ∆slyD::*Apra^R^ mutant** | | |
| *slyD-apra FD* | TAGTGAGTACACGGCTGCAGAATTCCGCTACAATCTGCGCCACTATTCTT |  |
| *slyD-apra* RV | TAGTGAGTACACGGCTGCAGAATTCCGCTACAATCTGCGCCACTATTCTT |  |
| *Apra-FD* | GGGTTCATGTGCAGCTCCA |  |
| *Apra-RV* | GGCAACACGTGGAGCGGATCG |  |
| **Construction of *misS-FLAG::Km and misU-FLAG::Km* strains** | | |
| *misS- flankA1* | TAGAAGTGGGGTGGGATTGACC |  |
| *misS- flankA2* | TTACTTATCGTCGTCATCCTTGTAATCATAAGAGCTTGAAATATTTCTCAA |  |
| *misS- flankB1* | TAGTACCTGGAGGGAATAATAAAGAATATAAAGGAATCAAAA |  |
| *misS- flankB2* | CTAGCGCACTCGCACACGATG |  |
| *misU- flankA1* | GAGATCATCGTGTGCGAGTGCG |  |
| *misU- flankA2* | TTACTTATCGTCGTCATCCTTGTAATCAATCGGTAACACCCTGATACT |  |
| *misU- flankB1* | GTTTTAGTACCTGGAGGGAATAATTTGTTTTGAACTTTTTAGGGG |  |
| *misU- flankB2* | CACTTTCCTAGCCACTTAGGGC |  |
| *FLAG-KAN FD* | GATTACAAGGATGACGACGATAAGTAAGAATTCGAGCTCGGTACCCGGG |  |
| *FLAG-KAN RV* | TTATTCCCTCCAGGTACTA |  |
| **Construction of the pILL2157 *misSU* plasmid** | | |
| *misS-SpeI FD* | GGACTAGTTTAAGGAGAACACTCATTTGTTACAACG | *Spe*I |
| *misU-SpeI RV* | GGACTAGTTCCTCAAATCGGTAACACCCTGATAC | *Spe*I |
| **Construction of the pILL2150 *slyD* and *slyD-∆Cter* plasmids** | | |
| *Ec.slyD FD* | CGCGGATCCgatgAAAGTAGCAA | *Bam*HI |
| *Ec.slyD RV* | CGgaattcGAACTATTCATGACC | *Eco*RI |
| *Ec. slyD∆C RV* | TGAATTCcgTGAAGCTAAAATTTCTTCTTC | *Eco*RI |
| **Constructions for the bacterial two hybrid screen** | | |
| **Cloning of *E. coli* *slyD* into pKT25 and of *H. pylori slyD* into pNKT25** | | |
| *Ec.slyD-KT25 FD* | CCCAAGCTTCCCATGCTCAGGAGAT | *Hin*dIII |
| *Ec.slyD-KT25 RV* | CGGAATTCTTAGTGGCAACCGCAACC | *Eco*RI |
| *Hp.slyD-NKT25 FD* | AAACTGCAGATATAGAGAAGCGATCAAG | *Pst*I |
| *Hp.slyD-NKT25RV* | AAAGAATTCGAACTATTCATGACCTTGCC | *Eco*RI |
| **Cloning of *E. coli* *fumA, E. coli* *fumB and E. coli* *fumC* into pUT18C** | | |
| *Ec.fumA-18C FV* | CTCTAGAGATGTCAAACAAACCCT | *Pst*I |
| *Ec.fumA-18C RV* | GGAATTCTTATTTCACACAGCGG | *Eco*RI |
| *Ec*.*fumB -18C FV* | GCTCTAGAGATGTCAAACAAACCCT | *Pst*I |
| *Ec*.*fumB -18C RV* | GGAATTCTTACTTAGTGCAGTTCGCG | *Eco*RI |
| *Ec.fumC* *-18C FV* | AAACTGCAGAATGAATACAGTACGCAGC | *Pst*I |
| *Ec.fumC* *-18C RV* | CGAGCTCTTAACGCCCGGCTTTC | *Sac*I |
| **Cloning of *iscA, iscU, iscS and sufS* into pUT18** | | |
| *iscA FD* | GGCACTGCAGGATGTCGATTACACTGAGCGACAG | *Pst*I |
| *iscA RV* | GGGGGTACCCGTCAAACGTGGAAGCTTTCGCCGCAAC | *Kpn*I |
| *iscU FD* | GGCACTGCAGGATGGCTTACAGCGAAAAAGTTATC | *Pst*I |
| *iscU RV* | GGGGGTACCCGTTATTTTGCTTCACGTTTGCTTTTATAGTCCG | *Kpn*I |
| *iscS FV* | GGCACTGCAGGATGAAATTACCGATTTATCTC | *Pst*I |
| *iscS RV* | GGGGGTACCCGTTAATGATGAGCCCATTCGATGCTGTTCAGATC | *Kpn*I |
| *sufS FV* | GGCACTGCAGGATGATTTTTTCCGTCGACAAAGTGC | *Pst*I |
| *sufS RV* | GGGGGTACCCGTTATCCCAGCAAACGGTGAATACGTTGCAGGCC | *Kpn*I |
| **Cloning of *H. pylori fumC, misS, misU, oorD and hemN* into pUT18C** | | |
| *fumC FD* | AAACTGCAGAATGCAATTTAGAATTGA |  |
| *fumC RV* | GGAATTCTCAAGCCTTAGGTCCG |  |
| *misS FD* | AAACTGCAGTATGTAAAATTTTAAGGAG |  |
| *misS RV* | AAGAATTCGAATAAGAGCTTGAAATATTTC |  |
| *misU FV* | AAACTGCAGTTAATAAAGAATATAAAGGAATC |  |
| *misU RV* | AAAGAATTCGAAATCGGTAACACCCTGATAC |  |
| *oorD FV* | AAACTGCAGAATGGCTAAAATGAGCGCT |  |
| *oorD RV* | GGAATTCTTATTTGTCTCTCCC |  |
| *hemN FV* | AAACTGCAGAATGCAAACCATTGATT |  |
| *hemN RV* | GGAATTCTCATAGCGTTTTACTGA |  |
| **RT-qPCR primers** | | |
| *ppk FD* | GCGTTAGTCGTTTATGGCGTTT |  |
| *ppk RV* | CGCTTAAAGGGTTGTAATTGCC |  |
| *misS FD* | ATCCCTGATGTGATGGTGGT |  |
| *misS RV* | CCAATTCCTTACTCGCTCCA |  |
| *misU FV* | GATCGCAAGCTCAGACATGA |  |
| *misU RV* | TCAGCGTTTTTCCCCAAATA |  |
